# Codon reassignment to facilitate genetic engineering and biocontainment in the chloroplast of *Chlamydomonas reinhardtii*

**DOI:** 10.1111/pbi.12490

**Published:** 2015-10-15

**Authors:** Rosanna E. B. Young, Saul Purton

**Affiliations:** ^1^ Algal Research GroupInstitute of Structural and Molecular BiologyUniversity College LondonLondonUK

**Keywords:** *Chlamydomonas reinhardtii*, chloroplast, microalgae, non‐sense suppression, transfer RNA

## Abstract

There is a growing interest in the use of microalgae as low‐cost hosts for the synthesis of recombinant products such as therapeutic proteins and bioactive metabolites. In particular, the chloroplast, with its small, genetically tractable genome (plastome) and elaborate metabolism, represents an attractive platform for genetic engineering. In *Chlamydomonas reinhardtii*, none of the 69 protein‐coding genes in the plastome uses the stop codon UGA, therefore this spare codon can be exploited as a useful synthetic biology tool. Here, we report the assignment of the codon to one for tryptophan and show that this can be used as an effective strategy for addressing a key problem in chloroplast engineering: namely, the assembly of expression cassettes in *Escherichia coli* when the gene product is toxic to the bacterium. This problem arises because the prokaryotic nature of chloroplast promoters and ribosome‐binding sites used in such cassettes often results in transgene expression in *E. coli*, and is a potential issue when cloning genes for metabolic enzymes, antibacterial proteins and integral membrane proteins. We show that replacement of tryptophan codons with the spare codon (UGG→UGA) within a transgene prevents functional expression in *E. coli* and in the chloroplast, and that co‐introduction of a plastidial *trnW* gene carrying a modified anticodon restores function only in the latter by allowing UGA readthrough. We demonstrate the utility of this system by expressing two genes known to be highly toxic to *E. coli* and discuss its value in providing an enhanced level of biocontainment for transplastomic microalgae.

## Introduction

The microalgal chloroplast has many advantages as a production platform for recombinant proteins and small molecules including low culturing costs, lack of toxins and ease of genetic manipulation. The presence of multiple copies of the chloroplast genome per cell and lack of gene silencing give the chloroplast an advantage over nuclear‐encoded transgene expression (Bock, 2015). The green alga *Chlamydomonas reinhardtii* is the most widely used for recombinant protein expression, with products such as vaccines, immunotoxins, therapeutics and industrial enzymes (reviewed by Rasala and Mayfield (2015) and Scaife *et al*. (2015)). Chloroplasts evolved from a cyanobacterial endosymbiont (Timmis *et al*., 2004) and many chloroplast genes in *C. reinhardtii* have retained bacterial features such as −35 and/or −10 promoter elements and 70S ribosome‐binding sequences. This is the case for the promoter and 5′ untranslated region (5′ UTR) of exon 1 of *psaA*, encoding a core subunit of photosystem I. The *psaA* promoter/5′ UTR is often used to drive robust expression of foreign genes in the *C. reinhardtii* chloroplast (Michelet *et al*., 2011; Specht and Mayfield, 2013; Young and Purton, 2014), but it cannot be used for proteins that are detrimental to *Escherichia coli* as they will be expressed during cloning in this host and will prevent successful production of the plasmid vector for subsequent transfer to the microalga. For example the PanDaTox database lists over 40 000 microbial genes that are predicted to be toxic to *E. coli* based on their failure to be propagated during genome sequencing projects (Amitai and Sorek, 2012). This lack of clonability can constrain the modification or introduction of biochemical pathways in *C. reinhardtii* for metabolic engineering due to alterations in carbon or nitrogen flux in *E. coli* or the generation of toxic intermediates, and may also prevent the cloning of genes for some antibacterial enzymes or integral membrane proteins.

Transfer RNAs (tRNAs) and their cognate aminoacyl‐tRNA synthetases together determine the amino acid sequence that is encoded by messenger RNA, so manipulation of these components can alter the genetic code. In the standard genetic code, 61 of the 64 RNA triplet codons are translated as amino acids whereas the remaining three (UAA, UAG and UGA) are stop signals at which release factors aid the termination of translation. The *C. reinhardtii* chloroplast genome uses this standard genetic code; however, DNA sequencing revealed that there is a strong preference for UAA as the stop codon with 65 of the 69 protein‐coding genes using this codon (Maul *et al*., 2002). The remaining four genes use UAG, and UGA is not used at all, although early genetic evidence demonstrates that it can function as a stop codon in the *C. reinhardtii* chloroplast. For example non‐photosynthetic mutants were isolated in which the chloroplast *rbcL* gene, encoding the large subunit of Rubisco, contained a TGG to TAG (amber) or TGA (opal) non‐sense mutation (Spreitzer *et al*., 1985). Chemical mutagenesis of the amber mutant, followed by selection for photosynthetic competence, produced a cell line in which the wild‐type *trnW*_*CCA*_ gene and a mutated version with an amber‐specific CUA anticodon coexisted as a heteroplasmic mix in the polyploid plastome, thus allowing both UGG and UAG codons to be translated as tryptophan (Yu and Spreitzer, 1992). A similar experiment using the opal mutant also produced heteroplasmic non‐sense suppressors but the genetic basis of the suppression was not characterized (Spreitzer *et al*., 1984). These results suggested that it would be possible to genetically engineer the *C. reinhardtii* chloroplast *trnW* to recognize amber, and possibly opal, codons instead of UGG.

Here, we address the challenge of cloning genes whose products are toxic to *E. coli* by exploiting the unused UGA codon to create a genetic system in which the gene of interest (GOI) is modified to carry opal mutations at one or more tryptophan codons (i.e. UGG to UGA), thereby preventing synthesis of the full‐length protein in either *E. coli* or the chloroplast. Translational read‐through is restored in the chloroplast, but not in *E. coli*, by combining the GOI with a plastidial *trnW* gene encoding a tRNA with a modified anticodon.

The existence of an unused codon in the *C. reinhardtii* chloroplast genetic code, together with our demonstration that it can be integrated into coding sequences and translated without the need to eliminate any plastidial release factors, provides opportunities for future genetic engineering of the microalgal chloroplast involving canonical or non‐canonical amino acids. The use of a non‐sense codon to interrupt the coding sequence also reduces the risk of transgenes being translated into full‐length proteins were they to spread to other organisms by horizontal gene transfer, thereby providing informational containment of the transgenes.

## Results

**Figure 1:**
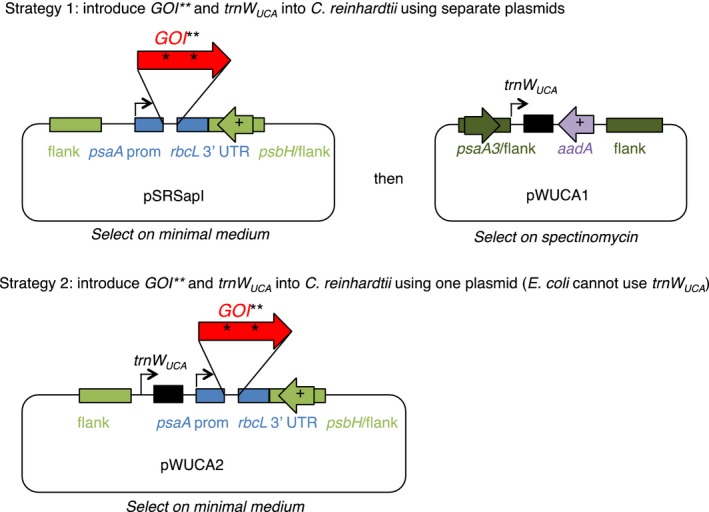
Strategy to clone genes into a chloroplast expression vector whilst preventing their expression in *Escherichia coli*. The gene of interest (*GOI*) is redesigned such that one or more tryptophan (TGG) codons are altered to TGA, indicated by asterisks. These changes can be integrated into the codon‐optimized gene design prior to ordering the synthetic gene and integrating it into the *Chlamydomonas reinhardtii* chloroplast genome. A tRNA gene based on the *C. reinhardtii* plastidial *trnW*, but with the anticodon sequence altered to recognize UGA, is also introduced (*trn* *W*_*UCA*_). This enables readthrough of the GOI in *C. reinhardtii*. Flanking regions amplified from chloroplast DNA allow targeted integration of the constructs into the chloroplast genome by homologous recombination; the target site is a neutral region downstream of either *psbH* or *psaA* exon 3, depending on the construct. The *psbH* gene can be used for selection in a *psbH* mutant recipient strain.

Our scheme for cloning genes that are toxic to *E. coli* into a *C. reinhardtii* chloroplast expression vector requires firstly a version of the gene with one or more TGG codons modified to TGA ‘stop’ codons, and secondly a synthetic tRNA gene to read the TGA codon/s as tryptophan. Strategies for introducing these two elements into *C. reinhardtii* are outlined in Figure 1. Plasmids used in this work are detailed in Table 1.

**Table 1:** Plasmids used in this work. All selection plates were incubated in the light except where pPsaA* was used. All synthetic protein‐coding genes were codon‐optimized for the *Chlamydomonas reinhardtii* chloroplast and encode a C‐terminal HA tag

Plasmid	Synthetic gene	Protein encoded by gene	Expected protein size incl. HA tag (kDa)	Integration site in *C. reinhardtii* chloroplast	Selection
pCD	*crCD*	Cytosine deaminase (Young and Purton, 2014)	49	Downstream of *psbH*	Minimal medium (*psbH* restored)
pCD**	*crCD* with 2 internal TGA stop codons	Cytosine deaminase	49 (if pWUCA1 also present)	Downstream of *psbH*	Minimal medium (*psbH* restored)
pWUCA1	*trnW*_*UCA*_	n/a	n/a	Downstream of *psaA* exon 3	Spectinomycin (*aadA* cassette)
pWUCA2	*trnW*_*UCA*_	n/a	n/a	Downstream of *psbH*	Minimal medium (*psbH* restored or psaA* translated, depending on recipient cell line)
pWUCA2‐CD**	*crCD* with 2 internal TGA stop codons + *trnW*_*UCA*_	Cytosine deaminase	49	Plasmid not used in *C. reinhardtii*	Plasmid not used in *C. reinhardtii*
pPsaA*	None; introduces TGG to TGA mutation at W693 in native *psaA* gene	Core protein of photosystem I	n/a	*psaA* exon 3 region	Spectinomycin (*aadA* cassette); incubate in dark
pSty	*Salmonella* Typhimurium phage gene SPN9CC_0043 with 1 internal TGA stop codon + *trnW*_*UCA*_	Endolysin (lyses *Escherichia coli*; Lim *et al*., 2014)	19	downstream of *psbH*	Minimal medium (*psbH* restored)
pSde	*Shewanella denitrificans* Sden_1266 with 2 internal TGA stop codons + *trnW*_*UCA*_	Hypothetical protein (very toxic to *E. coli*; Kimelman *et al*., 2012)	42	Downstream of *psbH*	Minimal medium (*psbH* restored)

### Mutation of two TGG codons to TGA codons within a transgene (*crCD*) prevents accumulation of CrCD protein in *Chlamydomonas reinhardtii*

The first set of experiments was carried out using *crCD* as a test gene. This is an *E. coli* cytosine deaminase gene optimized for the *C. reinhardtii* chloroplast as a negative selectable marker (Young and Purton, 2014) and was chosen as a test gene due to the stability of CrCD protein in the chloroplast, ease of detection by Western blotting (via an added HA epitope) and clear phenotype of sensitivity to 5‐fluorocytosine. CrCD is not toxic when expressed in *E. coli*, allowing appropriate control strains to be used.

**Figure 2:**
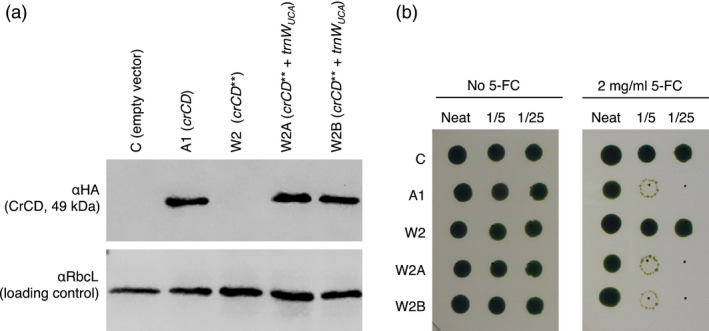
Introduction of a synthetic *trn* *W*_*UCA*_ gene into the *Chlamydomonas reinhardtii* chloroplast genome allows the expression of full‐length, active CrCD protein from the crCD** gene. (a) Western analysis. Equalized cell lysates were subjected to SDS‐PAGE and two identical blots were probed with antibodies as indicated on the left. (b) Growth tests demonstrating that cell lines A1, W2A and W2B contain active CrCD protein that leads to cell death on media containing 5‐fluorocytosine (5‐FC). *C. reinhardtii* cultures were adjusted to equal optical densities and spotted onto TAP agar containing no drug (left panel) or 5‐FC (right panel), with serial fivefold dilutions left to right. Plates were incubated under 50 μE/m^2^/s light for 10 days.

A chloroplast expression vector containing *crCD* under the control of the *C. reinhardtii psaA* exon 1 promoter (plasmid pCD, previously called pRY127d; (Young and Purton, 2014)) was modified so that two of the TGG codons, encoding tryptophan, were altered to TGA (Table S1). The resulting plasmid, pCD**, was used to transform *C. reinhardtii* TN72 (a non‐photosynthetic *psbH* mutant). The flanking region of pCD** contains an intact copy of *psbH*, so homologous recombination into the chloroplast genome restores phototrophic growth and allows the selection of transformants on minimal medium in the light. Transgene integration and homoplasmy across the approximately 80 copies of the chloroplast genome per cell was confirmed by PCR (Figure S1a and Table S2). As expected, the introduction of TGA codons into the *crCD* gene prevented the accumulation of full length CrCD protein as detected by immunoblotting with an antibody against the C‐terminal HA tag (cell line W2 in Figure 2a). This suggests that no tRNA in the *C. reinhardtii* chloroplast is able to recognize the UGA codon and insert an amino acid at the corresponding position in the peptide chain, so the chain terminates prematurely.

### Introduction of a synthetic *trnW*_*UCA*_ gene into the chloroplast genome allows the accumulation of full length, active CrCD protein and does not affect growth

There is a single codon for tryptophan in the standard genetic code (UGG) and a single copy of the tryptophan tRNA gene, *trnW*_*CCA*_, in the *C. reinhardtii* chloroplast genome (Cognat *et al*., 2013; Maul *et al*., 2002); see the PlantRNA database at http://plantrna.ibmp.cnrs.fr. A tRNA with a 5′‐UCA‐3′ anticodon would be expected to recognize UGA codons within the mRNA, such as those transcribed from the TGA codons that had been inserted into *crCD*. To test this, a version of the *C. reinhardtii* chloroplast *trnW* gene with 100 bp of its flanking sequence each side, which included its promoter, was synthesized with a mutated anticodon (CCA to UCA) and cloned into a chloroplast targeting vector to make plasmid pWUCA1 (Figure 1). The vector inserts the *trnW*_*UCA*_ gene downstream of *psaA* exon 3 in the chloroplast genome and contains an *aadA* spectinomycin resistance cassette. Homoplasmic transformants were recovered following selection on medium containing spectinomycin (Figure S1b and Table S2). The transformation of *C. reinhardtii* W2 with plasmid pWUCA1 was found to elicit the accumulation of full‐length CrCD protein (Figure 2a, cell lines W2A and W2B), demonstrating that the synthetic *trnW*_*UCA*_ gene is expressed and indeed recognizes the UGA codon. This also confirms that the 100 bp flanking sequences included around *trnW*_*UCA*_ were sufficient for its transcription and any subsequent 5′ and 3′ processing by RNaseP and other RNases. There was no observable difference in CrCD protein yield between W2A and a control *C. reinhardtii* cell line that had been transformed with pCD (Figure S2).

Cytosine deaminase (CrCD) normally converts cytosine to uracil but can also convert the synthetic compound 5‐fluorocytosine to a toxic product, 5‐fluorouracil. The activity of the CrCD protein made using the synthetic tRNA was demonstrated by the reduced growth of cell lines W2A and W2B on medium containing 5‐fluorocytosine (Figure 2b).

The expression of a synthetic tRNA that reassigns a stop codon sometimes slows the growth of the organism, presumably due to the lack of proper termination of endogenous proteins (Wang *et al*., 2014). As UGA is not used as a stop or sense codon in the *C. reinhardtii* chloroplast, we did not expect to see a growth defect in this case. Indeed, we found that the synthetic *trnW*_*UCA*_ gene expressed in the chloroplast had no detrimental effect on the growth rate of *C. reinhardtii* when tested under mixotrophic or phototrophic conditions at 25 °C (Figure S3).

### The tRNA and gene of interest can be introduced into the chloroplast in a single homologous recombination step

**Figure 3:**
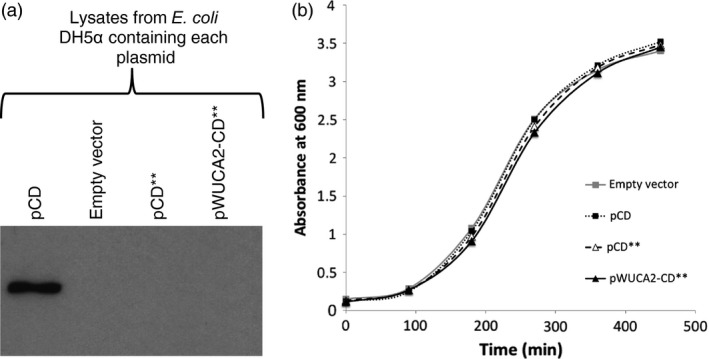
In *Escherichia coli*, the synthetic *Chlamydomonas reinhardtii* chloroplast *trn* *W*_*UCA*_ gene does not permit full length CrCD protein expression from the crCD** gene. (a) Western analysis using equalized lysates of *E. coli* DH5α carrying four different plasmids. The blot was probed with an αHA antibody to detect HA‐tagged CrCD protein. (b) Growth curve of *E. coli* DH5α carrying the four different plasmids.

The production of *C. reinhardtii* cell lines that express transgenes using the synthetic tRNA could be streamlined if both the transgene and tRNA gene were combined into one vector. This would require a single algal transformation step and would minimize the use of drug selection cassettes, especially if the restoration of *psbH* (i.e. growth on minimal medium) was used as the selection method. However, for this strategy to be useful for transgene containment and cloning toxic genes, *E. coli* must be unable to use the synthetic tRNA to translate the foreign protein. This was shown to be the case by inserting the *trnW*_*UCA*_ gene and its 100 bp flanks upstream of the *crCD* expression cassette in plasmid pCD**, generating pWUCA2‐CD**. No CrCD protein was detected in *E. coli* pWUCA2‐CD** lysates by immunoblotting with an anti‐HA antibody (Figure 3a), indicating that *E. coli* cannot use the synthetic tRNA to read through the UGA codons within the crCD** mRNA. This is more likely to be due to a lack of tRNA function or to competition with other factors than to a lack of tRNA transcription, as the *trnW*_*UCA*_ flank contains an exact bacterial consensus promoter (see Discussion and Appendix S1). The presence of a *trnW*_*UCA*_ plasmid did not affect the growth rate of *E. coli* (Figure 3b).

A new *trnW*_*UCA*_ chloroplast expression vector, pWUCA2, was then constructed into which a transgene can be cloned between the *psaA* exon 1 promoter/5′ UTR and the *rbcL* 3′ UTR using SapI and SphI restriction enzymes. The pWUCA2 plasmid carries the *trnW*_*UCA*_ gene upstream of this expression cassette (see Strategy 2 in Figure 1).

### The *trnW*_*UCA*_ gene rescues the mutation of an essential tryptophan codon to TGA in *psaA*

Tryptophan is the largest canonical amino acid and is the only one to carry an indole side‐chain. The double ring structure of its indole moiety is often involved in stacking interactions that are important for substrate binding and catalysis in some enzymes (Nakamura *et al*., 2013; Zhang *et al*., 2004a). To demonstrate that the synthetic trnW_UCA_ adds tryptophan rather than any other amino acid to the growing peptide chain, we carried out experiments on the *C. reinhardtii* chloroplast *psaA* gene, which encodes a core component of photosystem I (PSI).

The tryptophan at position 693 of PsaA is π‐stacked through the indole moiety with the bound phylloquinone cofactor (Boudreaux *et al*., 2001; Jordan *et al*., 2001). *Chlamydomonas reinhardtii* strains in which W693 has been substituted for another amino acid have a functional PSI complex but are highly sensitive to oxygen during phototrophic growth, possibly from the formation of free radical species (Purton *et al*., 2001).

**Figure 4:**
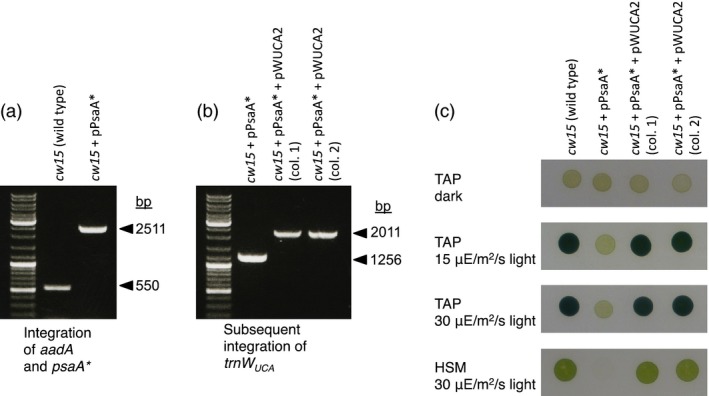
The mutation of a tryptophan codon that is essential for PsaA function in *Chlamydomonas reinhardtii* can be complemented by trnW_UCA_. (a) PCR on *C. reinhardtii* cell lines showing the homoplasmic integration of *aadA* downstream of *psaA* in the chloroplast genome, with the concurrent mutation of the *psaA* W693 codon to TGA (confirmed by sequencing). (b) PCR showing the homoplasmic integration of pWUCA2 into the *C. reinhardtii* pPsaA* cell line. Selection was directly for *trn* *W*_*UCA*_ to restore phototrophic growth by allowing the translation of psaA*. (c) Growth properties of the cell lines on media containing acetate (TAP) or minimal medium (HSM). *Chlamydomonas reinhardtii* cultures were adjusted to equal optical densities, spotted onto agar and incubated at 25 °C in the presence of oxygen.

We substituted the W693 TGG codon in *psaA* with TGA by homologous recombination (plasmid pPsaA* in Table 1), using an *aadA* spectinomycin resistance cassette for the selection of *C. reinhardtii* transformants. Homoplasmic integration of *aadA* downstream of *psaA* exon 3 was confirmed by PCR (Figure 4a), and the introduction of the non‐sense codon into *psaA* was confirmed by DNA sequencing (Figure S4). The resulting strain, *cw15* + pPsaA*, shows the loss of phototrophy and the light sensitivity typical of PSI‐deficient mutants (Figure 4c).

The *cw15* + pPsaA* cell line was transformed with pWUCA2, plating on minimal medium in the light under aerobic conditions. Although the intact *psbH* gene in pWUCA2 can be used for selection in a *psbH* mutant line such as TN72, the recipient cell line used here already has an intact *psbH* so this gene was merely being used as a homologous flanking region for integration of *trnW*_*UCA*_ into the chloroplast genome. Instead, selection was directly for trnW_UCA_ to translate full‐length, W693‐containing PsaA protein for restored photosynthesis, effectively using the tRNA gene as a positive selectable marker. Four out of seven *cw15* + pPsaA* + pWUCA2 colonies checked by PCR were homoplasmic for *trnW*_*UCA*_ after a single round of streaking out on minimal medium, demonstrating that selection was successful. We continued with transformants 1 and 2 (Figure 4b and Table S2); DNA sequencing of part of *psaA3* confirmed that they were not W693 TGA→TGG revertants (Figure S4). These cell lines can grow on minimal medium in the presence of oxygen (Figure 4c), contrasting with the oxygen‐sensitive phenotype of PsaA mutants that have other amino acids at position 693 (Purton *et al*., 2001).

The ability of trnW_UCA_ to complement the psaA* mutation indicates that the single nucleotide change in the anticodon loop from the natural tryptophan tRNA, trnW_CCA_, to the synthetic tRNA, trnW_UCA_, does not prevent the tryptophanyl tRNA synthetase from recognizing this as a tRNA to be charged with tryptophan.

### Genes whose products are toxic to *Escherichia coli* can be cloned using the synthetic tRNA system

The trnW_UCA_ cloning scheme using the combined vector (Figure 1) was tested using two genes whose products are known to be toxic to *E. coli*. The SPN9CC endolysin, from a *Salmonella* Typhimurium bacteriophage, has previously been shown to lyse *E. coli* (Lim *et al*., 2014). A codon‐optimized version of this endolysin gene was designed in which a single TGG codon was altered to TGA. This was cloned into pWUCA2 to make plasmid pSty. The second gene was a *Shewanella denitrificans* ORF identified from the PanDaTox database as unclonable in *E. coli* during genome sequencing. Further work by the compilers of the database showed that the ORF can be cloned under an inducible promoter in *E. coli* but that cells die upon induction of expression (Kimelman *et al*., 2012). A codon‐optimized version of this ORF was designed with two TGG to TGA mutations, and cloned into pWUCA2 to give plasmid pSde. DNA sequences for the two genes are given in Appendix S1.

**Figure 5:**
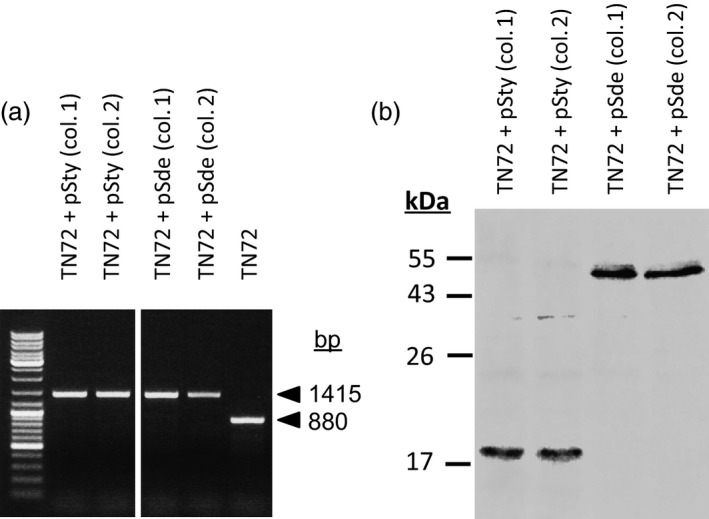
Use of the *trn* *W*_*UCA*_ system to clone and express genes in *C. reinhardtii* whose products are toxic to *Escherichia coli*. (a) PCR demonstrating homoplasmic integration of the transgenes into *C. reinhardtii* cell line TN72. (b) Western blot with anti‐HA antibody, demonstrating accumulation of the foreign proteins.

*Chlamydomonas reinhardtii* TN72 was transformed with pSty and pSde separately; homoplasmic integration of the transgenes and *trnW*_*UCA*_ was demonstrated by PCR (Figure 5a and Table S2). The accumulation of the SPN9CC endolysin and the *S. denitrificans* protein was demonstrated by immunoblotting with an anti‐HA antibody (Figure 5b).

### Stop codon usage in other microalgae

The selection of microalgal species for industrial biotechnology depends on many factors including the ease of genetic manipulation, growth rates, media costs, harvesting costs and, for some products, the lipid content. We surveyed the stop codon usage of 12 microalgal species for which chloroplast genome sequences were available to assess whether a similar approach to genetic code manipulation might be possible in species other than *C. reinhardtii* (Table S3). The chloroplast genomes ranged from 72 to 269 kb in size and were predicted to contain between 61 and 224 protein‐coding genes according to the annotations in the NCBI database. For each of the 12 genomes, UAA was the most frequently used stop codon and UGA was the least frequently used. As noted by Robbens *et al*. (2007), the chloroplast genome of *Ostreococcus tauri* does not use the UGA codon. *Lobosphaera* (*Parietochloris*) *incisa* also lacks UGA stop codons, and only a single putative gene (encoding a 45 amino acid hypothetical protein) has this stop codon in *Chlorella sorokiniana* (Table S3). However, the chloroplast transformation of these three species has yet to be reported. *Dunaliella salina*,* Euglena gracilis* and *Phaeodactylum tricornutum* each use the UGA stop codon three to four times in the chloroplast genome, so may be amenable to emancipation or dual use of this codon. Species in each of these three genera have been shown to have transformable chloroplast genomes (Doetsch *et al*., 2001; Georgianna *et al*., 2013; Xie *et al*., 2014).

## Discussion

The UGA codon is one of three triplet nucleotide codons used as stop signals in the standard genetic code. However, some species are known to have reassigned UGA to a sense codon. In the *C. reinhardtii* nucleus, UGA encodes stop or selenocysteine depending on the RNA context (Novoselov *et al*., 2002; Rao *et al*., 2003). Gracilibacteria translate this codon as glycine (Rinke *et al*., 2013), whilst UGA reassignments to tryptophan (known as Genetic Code 4) have been observed in mycoplasmas and their phages, in the bacterium *Candidatus* Hodgkinia cicadicola, and in some mitochondria (Ivanova *et al*., 2014; McCutcheon *et al*., 2009). The mitochondrion of the green alga *Pedinomonas minor* decodes both UGG and UGA as tryptophan using a single trnW (Turmel *et al*., 1999). These examples set a precedent for the reassignment of UGA in the *C. reinhardtii* chloroplast.

The recognition of more than one codon sequence by an anticodon (‘wobble’) involves post‐transcriptional modifications of the tRNA by one or more enzymes (Crick, 1966; El Yacoubi *et al*., 2012). Unmodified uridine in the wobble position of an anticodon (U_34_) binds only to adenosine in the third position of the codon (Agris *et al*., 2007); in the case of the synthetic *C. reinhardtii* chloroplast trnW_UCA_ this would mean that only the codon UGA would be recognized, as desired. U_34_ modifications in naturally occurring trnW_UCA_ include 5‐taurinomethyluridine in *Bos taurus* mitochondria and 5‐carboxymethylaminomethyluridine in *Tetrahymena thermophila* mitochondria (see Modomics database at http://modomics.genesilico.pl). U_34_ modifications can allow the anticodon to recognize certain other bases in the codon's wobble position, but the relationship is rather complex and not fully understood (Agris *et al*., 2007). We have not determined whether U_34_ in the synthetic tRNA is modified, but two findings indirectly suggest that wobble may not occur. First, the heteroplasmic nature of *C. reinhardtii* mutants that suppress a TGG→TGA mutation in *rbcL* (Spreitzer *et al*., 1985) suggests that a homoplasmic CCA→UCA mutation in the endogenous trnW anticodon would be lethal due to the prevention of UGG translation. Second, pleiotropic effects might be expected if our synthetic tRNA could insert tryptophan in native proteins in response to UGU or UGC cysteine codons, but no change in growth rate was observed in *C. reinhardtii* cell lines containing the tRNA.

Peptide chain release factors specifically recognize stop codons and can antagonize attempts to reassign these to sense codons. However, we found no observable difference in the level of CrCD protein accumulation between *C. reinhardtii* cell lines with an intact *crCD* gene or crCD** + *trnW*_*UCA*_ (Figure S2). This suggests that either there is no release factor that recognizes UGA codons in the *C. reinhardtii* chloroplast or such a factor is outcompeted successfully by trnW_UCA_. In eubacteria and the *Arabidopsis thaliana* chloroplast, release factor PrfA (RF1) recognizes UAA and UAG codons whilst PrfB (RF2) recognizes UAA and UGA; both trigger peptidyl‐tRNA hydrolysis. Since neither the chloroplast nor mitochondria in *C. reinhardtii* use UGA as a stop (or sense) codon, PrfB should not be required, but nevertheless a *prfB* orthologue (locus Cre01.g010864) is present in the nuclear genome. It is not clear from signal peptide analysis whether the resulting PrfB protein would be targeted to the chloroplast, mitochondria or both; the cytosol has its own eukaryotic release factor system. The motifs required for PrfB function and specificity are well studied in other organisms (Frolova *et al*., 1999; Ito *et al*., 2000; Johnson *et al*., 2011; Wilson *et al*., 2000). The stop codon recognition motif SPF, peptide release motif GGQ and essential Ser246 residue are all intact in the *C. reinhardtii* PrfB orthologue, although the predicted N‐terminus is dissimilar to those of the *E. coli* and *A. thaliana* PrfB proteins. *Chlamydomonas reinhardtii* may eventually lose the *prfB* gene, as has happened in *Candidatus* Hodgkinia cicadicola (McCutcheon *et al*., 2009). Alternatively it may be retained if it is required for efficient UAA termination or has been adapted and recruited to stabilize particular RNA transcripts, as is the case for PrfB3 in *A. thaliana* (Stoppel *et al*., 2011).

The synthetic tRNA did not allow detectable readthrough of UGA codons in *E. coli*. This observation allows the gene of interest and *trnW*_*UCA*_ to be combined into a single vector and also reduces the risk that transgenes would be translatable if they spread to other organisms by horizontal gene transfer. The two main factors likely to contribute to this lack of readthrough in *E. coli* are competition with the release factor PrfB and differences in the mechanism of specific tRNA function between *E. coli* and the *C. reinhardtii* chloroplast. Regarding the latter factor, the strongest recognition elements for the aminoacylation of *E. coli* trnW with tryptophan are the discriminator base G73, which is conserved across prokaryotic (but not eukaryotic) trnW, and the anticodon CCA (Himeno *et al*., 1991; Hughes and Ellington, 2010). The *C. reinhardtii* chloroplast trnW and its synthetic trnW_UCA_ counterpart do contain the G73 base. However, since the synthetic trnW_UCA_ necessarily carries a mutated anticodon, the *E. coli* tryptophanyl‐tRNA synthetase may not recognize it to be charged with tryptophan. Differences in the synthesis of the tRNA 3′ acceptor stem may also contribute to a lack of tRNA transferability between chloroplasts and *E. coli*: this trinucleotide sequence (also CCA) is included in tRNA genes in *E. coli*, whereas in the *C. reinhardtii* chloroplast and cyanobacteria it is added post‐transcriptionally by a nucleotidyltransferase enzyme (Schmidt and Subramanian, 1993; Xiong and Steitz, 2006).

An alternative strategy for cloning genes that are toxic to *E. coli* into chloroplast expression vectors was demonstrated by Oey *et al*. (2009), who inserted bacterial transcription termination signals between a selectable marker gene and a downstream endolysin gene. After cloning in *E. coli* and transformation of tobacco, the selectable marker gene and termination signals were subsequently removed by Cre‐*loxP* recombination to enhance endolysin expression. Whilst this strategy worked well for the gene tested and is a good compromise for genetic systems that lack a spare codon, there was a low level of leaky expression in *E. coli* so it would not be suitable for proteins that are highly toxic to this bacterium. In contrast, with the trnW_UCA_ strategy we did not detect any CrCD protein in the *E. coli* pWUCA2‐CD** cell line. If necessary, any leaky translation could be reduced further by increasing the number of TGA codons in the gene, with the maximum being the number of tryptophan residues in the protein.

Another approach to reduce the expression of bactericidal proteins during cloning is to culture the *E. coli* at a lower temperature, which increases plasmid supercoiling and reduces transcription of the transgene, at least when plant *rrn* and *psbA* promoters are used (Madesis *et al*., 2010). Due to the differential sensitivity of plastid promoters to topology (Stirdivant *et al*., 1985), the efficacy of this strategy is likely to vary between chloroplast expression vectors. Antimicrobial peptides, which would be lethal to *E. coli* if expressed on their own, are often expressed as fusion proteins to temporarily mask their function when this bacterium is used as an expression platform or cloning host (Lee *et al*., 2011; Li, 2009). This is an effective strategy but requires the extra processing step of protease cleavage, adding to the time and cost of protein production and making it inappropriate for the manipulation of metabolic pathways.

Genetic code manipulation can be used to introduce non‐canonical amino acids into certain positions within proteins *in vivo*, with applications including altering enzyme properties, enabling chemical modifications and providing trophic biocontainment by making an organism dependent on unnatural amino acids (Ravikumar and Liu, 2015). For example Zhang *et al*. (2004b) adapted the *Bacillus subtilis* tryptophanyl tRNA and its cognate synthetase to incorporate 5‐hydroxytryptophan in response to the opal stop codon UGA in mammalian cells. This required altering the tRNA anticodon sequence and a single amino acid in the active site of the synthetase. 5‐hydroxytryptophan has unique spectral properties and can be used to study protein structure and function. As long as non‐canonical amino acids can be taken up into the chloroplast from the growth medium, the spare UGA codon in the *C. reinhardtii* chloroplast genetic code could be used to encode such an amino acid. According to the Codon Usage Database (www.kazusa.or.jp) there are no spare codons in the plastomes of the model higher plants *Arabidopsis thaliana*,* Nicotiana tabacum* or *Zea mays*, but the reassignment of existing codons such as UGA or the use of quadruplet codons may be possible, if less efficient. Indeed, non‐canonical amino acids have been engineered into bacteria, yeast and mammalian cells despite the lack of spare codons in these organisms (Niu *et al*., 2013; Wang and Wang, 2012).

A typical *E. coli* genome contains 2765 TAA (ochre), 321 TAG (amber) and 1249 TGA (opal) stop codons (www.kazusa.or.jp, Codon Usage Database). Amber is the rarest codon in *E. coli* and has been successfully assigned to encode non‐canonical amino acids using transgenic orthogonal aminoacyl‐tRNA synthetase/tRNA pairs. In standard synthetic *E. coli* amber suppressor lines, UAG is still used for translation termination in many endogenous genes, and recent studies show that the cells evolve to counteract amber suppression by inserting transposons into the new aminoacyl‐tRNA synthetase gene and decreasing plasmid copy number (Wang *et al*., 2014). Growth rates are also decreased. These issues could hinder the development of stable, fast‐growing *E. coli* amber suppressor lines for industrial use. The generation of an *E. coli* strain with a truly emancipated amber codon for genetic engineering purposes involved the replacement of all 321 TAG stop codons in the genome with TAA, then the deletion of *prfA* encoding release factor 1, to prevent competition with the transgenic tRNA; unfortunately this strain has a 60% increased doubling time (Johnson *et al*., 2012; Lajoie *et al*., 2013).

In contrast, the *C. reinhardtii* chloroplast genome uses ochre, amber and opal stop codons 65, four and zero times respectively. This reflects the considerably smaller gene content of the chloroplast genome: most proteins in *C. reinhardtii* are encoded by the nuclear genome, including many chloroplast‐targeted proteins such as some components of the photosynthetic machinery (Harris *et al*., 2009). The genetic isolation of the plastidial translation system should enable its manipulation independently of the nuclear and mitochondrial genomes. Although UGA is the only codon that is completely absent from protein‐coding genes in the *C. reinhardtii* chloroplast genome, several other codons are rarely used and are potential targets for reassignment if several different non‐canonical amino acids were required in a single cell line. As well as the low number of amber (UAG) codons mentioned above, there are only six instances of CGG and 14 of CUC. The finding that the *C. reinhardtii* chloroplast UGA codon can be efficiently assigned simply with the addition of a modified tRNA gene provides a starting point for more advanced genetic code manipulation in microalgae.

## Experimental procedures

### *Chlamydomonas reinhardtii* strains and growth conditions

For experiments using pPsaA*, the cell wall‐deficient strain *C. reinhardtii cw15* was used as the recipient. For all other experiments, the recipient strain was *C. reinhardtii* TN72 (Young and Purton, 2014), which is a *cw15 psbH*‐deletion mutant. Cell lines were maintained on Tris‐acetate phosphate (TAP) plates with 2% agar (Harris *et al*., 2009) and were cultured for growth tests and Western analysis in flasks containing 20 mL TAP, shaking at 120 rpm and 25 °C. Where required, optical density was measured at 750 nm using a spectrophotometer.

### Plasmid construction

A synthetic *trnW*_*UCA*_ gene was designed by taking the *C. reinhardtii* chloroplast *trnW* gene sequence with 100 nt flanking DNA on each side (i.e. Genbank accession number BK000554.2, position 17481 to 17207; Appendix S1), altering the anticodon from CCA to TCA, and adding MluI restriction sites at both ends of the fragment for cloning. The DNA was synthesized as a linear fragment by Integrated DNA Technologies (Coralville, IA, USA) and cloned into the MluI site in pBev1 (Hallahan *et al*., 1995) to make pWUCA1, or into the MluI site in pSRSapI (Young and Purton, 2014) to make pWUCA2. The sequences of pWUCA1 and pWUCA2 are given in Appendix S1. Restriction enzymes were purchased from New England Biolabs (Ipswich, MA). Plasmids were cloned by the transformation of chemically competent *E. coli* DH5α using ampicillin selection (Sambrook and Russell, 2001) and extracted by alkaline lysis (Sambrook and Russell, 2001) or with a QIAfilter Plasmid Midi kit (Qiagen, Venlo, The Netherlands).

To make the pCD** construct, two TGG→TGA mutations were introduced into plasmid pCD/pRY127d (Young and Purton, 2014) by one‐step isothermal assembly using three PCR products (Gibson *et al*., 2009); primers are listed in Table S1 and DNA was amplified using Phusion High‐Fidelity DNA Polymerase (Thermo Scientific, Waltham, MA) according to the manufacturer's instructions. The mutations correspond to tryptophans W21 and W147 of the 436 aa CrCD protein. This is the first and sixth of the seven tryptophan residues in CrCD.

To make the pPsaA* construct, a TGG→TGA mutation at amino acid position W693 was introduced into the *psaA* exon 3 ORF in the plasmid pBev1 (Hallahan *et al*., 1995) by one‐step isothermal assembly of a single PCR product whose ends overlapped by 22 bp; primer sequences are given in Table S1. The DNA sequence of pPsaA* is given in Appendix S1.

Modified versions of the *Salmonella* Typhimurium bacteriophage SPN9CC endolysin and *Shewanella denitrificans* Sden_1266 genes were synthesized by Eurofins Genomics (Ebersberg, Germany) and Integrated DNA Technologies, respectively; see Appendix S1. They were each cloned into pWUCA2 using SapI and SphI restriction enzymes, placing them under a *psaA* exon 1 promoter. The resulting plasmids (pSty and pSde) are described in Table 1.

### *Chlamydomonas reinhardtii* transformation

Transformation was carried out using the glass bead vortex method as described in Young and Purton (2014), with selection on high‐salt minimal medium for constructs that restore *psbH* or PsaA accumulation and selection on TAP + 100 μg/mL spectinomycin for constructs containing an *aadA* spectinomycin resistance cassette. Colonies were checked for homoplasmy of the insertion by PCR using the primers shown in Table S2 and Phusion Polymerase (see above). PCR products were analysed on 1% agarose gels alongside GeneRuler DNA Ladder Mix (Thermo Scientific).

### Western blot analysis

10 mL mid‐log phase *C. reinhardtii* cultures grown under 90 μE/m^2^/s light were harvested for Western blot analysis of proteins. Preparation of lysates, SDS‐PAGE gels, blotting onto a nitrocellulose membrane and incubation in the primary αHA or αRbcL antibody were performed as described previously (Young and Purton, 2014) except that αHA was prepared in TBS with 0.1% Tween (TBS‐T) and 0.5% milk. Blots were then incubated for 1 h in the secondary antibody, goat αrabbit Dylight 800 (Thermo Scientific) diluted 1:25 000 in TBS‐T and 0.5% milk, washed in TBS‐T and imaged with an Odyssey Fc Imaging System (LI‐COR, Lincoln, NE) at 800 nm.

For the *E. coli* Western blot, strains were grown in LB with 100 μg/mL ampicillin overnight at 37 °C. Optical densities were measured at 600 nm then cultures were pelleted and resuspended in sample buffer to equal densities as described in the Mini‐PROTEAN Tetra Cell manual (Bio‐Rad, Hercules, CA). The protocol was then continued exactly as for the *C. reinhardtii* Western blots described in Young and Purton (2014). Briefly, samples were boiled, loaded onto a 15% acrylamide gel, blotted onto a nitrocellulose membrane and probed with αHA primary antibody and ECL αrabbit IgG HRP‐linked secondary antibody, with detection via chemiluminescence.

### 5‐fluorocytosine sensitivity test

Liquid *C. reinhardtii* cultures grown in TAP medium for 48 h were adjusted to an optical density of 0.4 at 750 nm. 5 μL was spotted onto TAP plates containing 2% agar and either no drug or 2 mg/mL 5‐fluorocytosine (Sigma‐Aldrich, St. Louis, MO, USA). Plates were incubated under 50 μE/m^2^/s light at 25 °C for 10 days.

### *Escherichia coli* growth curve

5 mL LB broths containing 100 μg/mL ampicillin were inoculated with overnight *E. coli* DH5α cultures containing each plasmid so that the starting absorbance at 600 nm was 0.1. Cultures were incubated at 37 °C with shaking, and the absorbance at 600 nm was measured every 90 min using a spectrophotometer.

## Supporting information


**Figure S1** Homoplasmy PCR for *crCD* strains.
**Figure S2** Western blot of CrCD protein levels in *Chlamydomonas reinhardtii* cell lines.
**Figure S3** *Chlamydomonas reinhardtii* growth curves with and without trnW_UCA_.
**Figure S4** Sequence confirmation of *psaA* codon alteration.
**Table S1** Primers used in the construction of plasmids.
**Table S2** Primers used to confirm homoplasmic integration of transgenes.
**Table S3** Stop codon distribution in microalgae.
**Appendix S1** DNA and amino acid sequences.
