# Supplementary material for: Codon reassignment to facilitate genetic engineering and biocontainment in the chloroplast of Chlamydomonas reinhardtii
Source: Plant Biotechnol J. 2015 Oct 15;14(5):1251–60. doi: 10.1111/pbi.12490 (PMC5102678; doi:10.1111/pbi.12490)
Supplement: Supplementary file 1 — Figure S1 Homoplasmy PCR for crCD strains. Figure S2 Western blot of CrCD protein levels in Chlamydomonas reinhardtii cell lines. Figure S3 Chlamydomonas reinhardtii growth curves with and without trnWUCA. Figure S4 Sequence confirmation of psaA codon alteration. Table S1 Primers used in the construction of plasmids. Table S2 Primers used to confirm homoplasmic integration of transgenes. Table S3 Stop codon distribution in microalgae. Appendix S1 DNA and amino acid sequences. [file PBI-14-1251-s001.docx]

**Supporting information**

**Codon reassignment to facilitate genetic engineering and biocontainment in the chloroplast of *Chlamydomonas reinhardtii***

Rosanna E. B. Young and Saul Purton

**Figure S1** PCR demonstrating homoplasmic integration of transgenes into the *C. reinhardtii* chloroplast genome. Primers are listed in Table S2.

(a) Integration of pCD** into strain TN72. W1 and W2 are two independent transformants. C is an empty-vector positive control strain.

(b) Integration of pWUCA1 into strain W2, introducing *trnW_UCA_* into a neutral region of the chloroplast genome next to *psaA3*. Three independent transformants are shown.


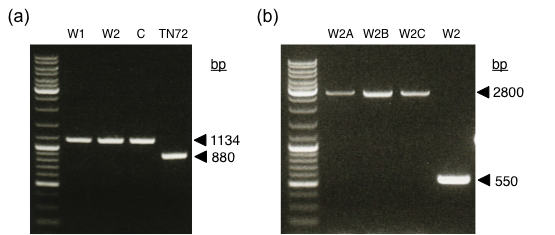


**Figure S2** SDS-PAGE of whole cell lysates for *C. reinhardtii* strains containing an intact *crCD* gene or a combination of *crCD*** with *trnW_UCA_*.

TAP cultures of the two strains were set up with a starting density of OD_750_ = 0.4 and grown for 24 h, reaching an OD_750_ of 1.2-1.3. Cells were pelleted and resuspended in loading buffer to equal optical densities. The upper part of the gel was blotted onto membrane, which was then probed with an αHA antibody to detect HA-tagged CrCD protein. The lower part of the gel was stained with Coomassie to confirm equal loading of the two strains. The left hand lane contains PageRuler Prestained Protein Ladder (Life Technologies): the bands visible on the blot are (from top) 170, 130, 100, 55, 40 and 35 kDa.


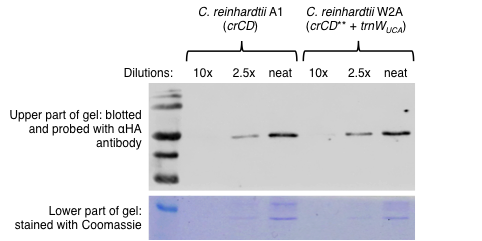


**Figure S3** Growth curves of *C. reinhardtii* cell lines with and without the synthetic tRNA gene, *trnW_UCA_*. All cultures were grown at 25 °C and shaken at 120 rpm under 90 μE/m^2^/s light. Optical density was measured at 750 nm.

(a) Mixotrophic growth: cells were grown in 20 ml TAP (acetate-containing) medium. Error bars show ± standard deviation of three cultures per cell line.

(b) Phototrophic growth: cells were grown in 20 ml HSM (minimal) medium.


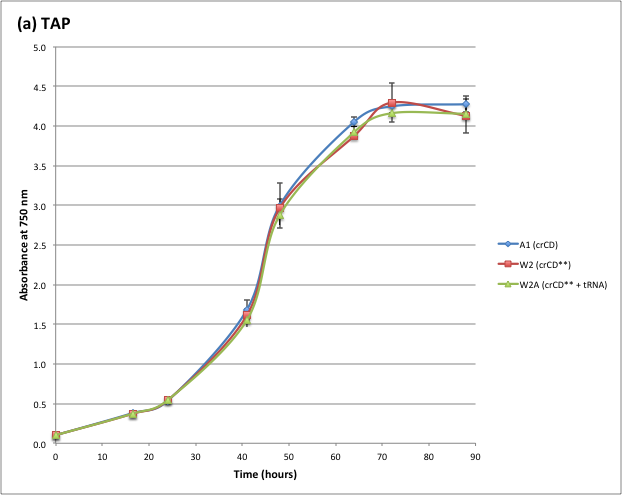


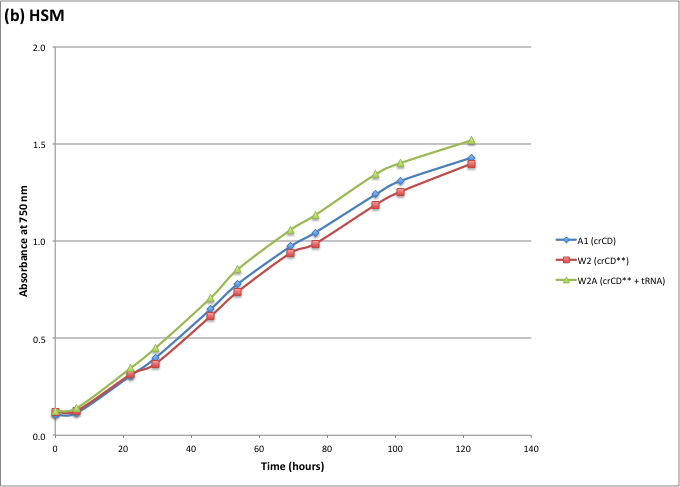


**Figure S4** Confirmation of *psaA* exon 3 codon alteration in *C. reinhardtii* cell lines by DNA sequencing. The W693 codon was altered from TGG to TGA by genetic engineering to create the *cw15* + pPsaA* cell line. When this mutant was transformed with the pWUCA2 plasmid, encoding *trnW_UCA_*, phototrophy was restored due to the readthrough of the TGA codon in *psaA**.


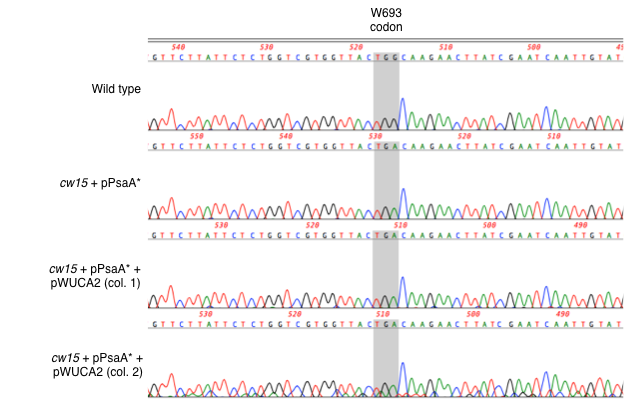


**Table S1** Primers used in the construction of plasmids.

| **Purpose** | **Reaction number** | **Primer sequences 5’ to 3’** |
| --- | --- | --- |
| Construction of pCD**: introduce 2 TGG🡺TGA mutations into pCD (pRY127d) by 3-part Gibson assembly (split backbone in AmpR gene to facilitate PCR) | PCR 1 (0.4 kb) | AAGGTTTATGACAAATTCACTTACAAGAC |
|  |  | GTAAGTCAATTCATGGAGCTACTTCTTG |
|  | PCR 2 (2.9 kb) | AGTGAATTTGTCATAAACCTTCTTCACC |
|  |  | GCCTTCCTGTTTTTGCTCA (in AmpR gene) |
|  | PCR 3 (4.2 kb) | ACTTTCACCAGCGTTTCTG (in AmpR gene) |
|  |  | TAGCTCCATGAATTGACTTACAAATTGC |
| Construction of pPsaA*: introduce W693 TGG🡺TGA mutation into pBev1 by 1-part Gibson assembly | PCR 1 (9.3 kb) | GTGGTTACTGACAAGAACTTATCGAATC |
|  |  | ATAAGTTCTTGTCAGTAACCACGACCAGA |
|  |  |  |
|  |  |  |

**Table S2** Primers used to confirm homoplasmic integration of transgenes into *C. reinhardtii.*

| **Figure** | **Plasmid used for transformation** | **Primer sequences 5’ to 3’** | **Product size for parental strain (bp)** | **Product size if homoplasmic integration (bp)** |
| --- | --- | --- | --- | --- |
| S1a | pCD** | GTCATTGCGAAAATACTGGTGC | 880 | 1134 |
|  |  | CGGATGTAACTCAATCGGTAG |  |  |
|  |  | ATAGGCTCTTCTCATGGATTTCTCCTTATAATAAC |  |  |
| S1b | pWUCA1 | agcacgtatcatttctgtagg | 550 | 2800 |
|  |  | GGGAAAGAGGGACTTGAAC |  |  |
| 4a | pPsaA* | agcacgtatcatttctgtagg | 550 | 2511 |
|  |  | GGGAAAGAGGGACTTGAAC |  |  |
| 4b | pWUCA2 | GTCATTGCGAAAATACTGGTGC | 1256 | 2011 |
|  |  | GCAACAGGAACTTCTAAAGC |  |  |
| 5 | pSty or pSde | GTCATTGCGAAAATACTGGTGC | 880 | 1415 |
|  |  | CGGATGTAACTCAATCGGTAG |  |  |
|  |  | ATAGGCTCTTCTCATGGATTTCTCCTTATAATAAC |  |  |

**Table S3** Stop codon distribution in microalgae.

****Compiled from annotated chloroplast genomes in Genbank. ‘No. of CDS’ is the number of protein-coding genes annotated in Genbank for each genome as of November 2014. The number of annotated TAA, TAG and TGA stop codons is listed. For comparison, the chloroplast genome of *Arabidopsis thaliana* Columbia is 154,478 bp long with 49 TAA, 19 TAG and 12 TGA stop codons according to Sato et al. (1999) *DNA Res* **6**, 283-290.

**Appendix S1** DNA and amino acid sequences.

**1. Cytosine deaminase gene used in plasmid pCD****

**(a) DNA sequence of *crCD*** gene, with TGG🡺TGA codon alterations in green.**

ATGTCTAACAACGCTTTACAAACAATTATTAACGCTCGTTTACCAGGTGAAGAAGGTTTA

TGACAAATTCACTTACAAGACGGTAAAATTTCAGCTATTGATGCTCAATCTGGTGTAATG

CCAATTACTGAAAACTCTTTAGATGCTGAACAAGGTTTAGTTATTCCACCATTCGTTGAA

CCACACATTCACTTAGATACTACACAAACAGCTGGTCAACCAAACTGGAACCAATCAGGT

ACTTTATTTGAAGGTATTGAGCGTTGGGCTGAACGTAAAGCTTTATTAACACACGACGAC

GTTAAACAACGTGCTTGGCAAACATTAAAATGGCAAATTGCTAACGGTATTCAACACGTA

CGTACTCACGTAGACGTTTCTGATGCTACTTTAACAGCTTTAAAAGCTATGTTAGAAGTT

AAACAAGAAGTAGCTCCATGAATTGACTTACAAATTGCTGCTTTCCCACAAGAAGGTATT

TTATCATACCCAAACGGTGAAGCTTTATTAGAAGAAGCTTTACGTTTAGGTGCTGATGTT

GTTGGTGCTATTCCACACTTCGAATTTACACGTGAATATGGTGTTGAATCTTTACACAAA

ACATTTGCTTTAGCTCAAAAATATGATCGTTTAATTGATGTTCACTGTGACGAAATTGAT

GACGAACAATCACGTTTCGTTGAAACAGTAGCTGCTTTAGCTCACCACGAAGGTATGGGT

GCTCGTGTTACTGCTTCACACACTACAGCTATGCACTCTTACAACGGTGCTTACACTTCT

CGTTTATTCCGTTTATTAAAAATGTCTGGTATTAACTTCGTTGCTAACCCATTAGTAAAC

ATTCACTTACAAGGTCGTTTCGATACTTACCCAAAACGTCGTGGTATTACACGTGTTAAA

GAAATGTTAGAATCAGGTATTAATGTTTGTTTTGGTCACGACGACGTTTGTGGTCCTTGG

TACCCTTTAGGTACTGCTAACATGTTACAAGTTTTACACATGGGTTTACACGTATGTCAA

TTAATGGGTTACGGTCAAATTAACGACGGTTTAAACTTAATTACTCACCACTCTGCTCGT

ACTTTAAACTTACAAGACTACGGTATTGCTGCTGGTAACTCAGCTAACTTAATTATTTTA

CCAGCTGAAAACGGTTTCGATGCTTTACGTCGTCAAGTTCCAGTACGTTACTCAGTTCGT

GGTGGTAAAGTTATTGCTTCAACTCAACCAGCTCAAACAACTGTTTATTTAGAACAACCA

GAAGCTATTGACTACAAACGTTACCCATACGATGTTCCAGATTACGCTTAATAA

**(b) Translated sequence of *crCD***.**

Tryptophans W21 and W147 that were mutated from TGG to TGA are shown in green. HA tag is shown in red. Residues in turquoise show where the synthetic CrCD enzyme differs from natural *E. coli* CodA for improved substrate binding; see Young and Purton (2014) *Plant J* **80**, 915-925.

MSNNALQTIINARLPGEEGLWQIHLQDGKISAIDAQSGVMPITENSLDAEQGLVIPPFVEPHIHLDTTQTAGQPNWNQSGTLFEGIERWAERKALLTHDDVKQRAWQTLKWQIANGIQHVRTHVDVSDATLTALKAMLEVKQEVAPWIDLQIAAFPQEGILSYPNGEALLEEALRLGADVVGAIPHFEFTREYGVESLHKTFALAQKYDRLIDVHCDEIDDEQSRFVETVAALAHHEGMGARVTASHTTAMHSYNGAYTSRLFRLLKMSGINFVANPLVNIHLQGRFDTYPKRRGITRVKEMLESGINVCFGHDDVCGPWYPLGTANMLQVLHMGLHVCQLMGYGQINDGLNLITHHSARTLNLQDYGIAAGNSANLIILPAENGFDALRRQVPVRYSVRGGKVIASTQPAQTTVYLEQPEAIDYKRYPYDVPDYA

**2. Sequence of the synthetic *trnW_UCA_* gene with 100 bp flanks, as used in plasmids pWUCA1 and pWUCA2.**

Colours represent the tRNA sequence (green), anticodon (blue), and MluI sites used for cloning (red). The -10 and -35 consensus promoter elements are in bold.

tcgatgc**ACGCGT**taacccatgattaacaactatatcaataaaatcaatttgtagtgaaatactctga**ttgaca**ttaaaataataccatgataaaaat**tataat**aacaaattttacgtccttagttcagtcggtagaacgcaggttt**tca**aaacctgatgtcgtgggttcaattcctacagggcgtgtttttcctaatgtactttgttgtaaaagtggctggtttaacctttttaggtttcggattgaacaataatggcagttaagagtcactaaagctgctgtatag**ACGCGT**tcgatgc

**3. Endolysin SPN9CC_0043 From phage from *Salmonella* Typhimurium**

The natural gene is described in Lim *et al*. (2014) *J Microbiol Biotechnol* **24**, 803-811; accession number YP_006383882. For the present paper, a codon optimised version of this endolysin gene was designed in which a single TGG codon was altered to TGA (green). This was cloned into pWUCA2 using SapI and SphI restriction sites (yellow) to make plasmid pSty. The HA tag sequence is shown in red.

**(a) DNA sequence**

GCTCTTCTATGGCTATGAGTCCAGCTTTACGTAACTCAGTAATTGCTGCTATTTCTGGTGGTGCTATTGCTATTGCTTCAGTATTAATTACTGGTCCAGGTGGTAACGACGGTTTAGAAGGTGTTCGTTACAAACCATACAAAGACGTAGTAGGTGTTTTAACAGTTTGTTACGGTCACGTAGGTAAAGATATTATGTTAGGTAAAACATACACAGAAGCTGAATGTGAAGCTTTATTAAACAAAGACTTAGCTACTGTAGCTCGTCAAATTAACCCATACATTAAAGTAGATATTCCAGAAACTACACGTGGTGCTTTATACAGTTTTGTTTACAACGTTGGTGCTGGTAACTTCCGTACTTCAACTTTATTACGTAAAATTAACCAAGGTGATATTAAAGGTGCTTGTGACCAATTACGTCGTTGAACTTACGCTGGTGGTAAACAATGGAAAGGTTTAATGACACGTCGTGAAATTGAACGTGAAGTTTGTTTATGGGGTCAACAATACCCATACGATGTTCCAGATTACGCTTAATAAGCATGC

**(b) Translated sequence**

MAMSPALRNSVIAAISGGAIAIASVLITGPGGNDGLEGVRYKPYKDVVGVLTVCYGHVGKDIMLGKTYTEAECEALLNKDLATVARQINPYIKVDIPETTRGALYSFVYNVGAGNFRTSTLLRKINQGDIKGACDQLRRWTYAGGKQWKGLMTRREIEREVCLWGQQYPYDVPDYA

**4. *Shewanella denitrificans* Sden_1266 (hypothetical protein)**

A codon optimised version of this gene was designed in which two TGG codons were altered to TGA (green). This was cloned into pWUCA2 using SapI and SphI restriction sites (yellow) to make plasmid pSde. The HA tag sequence is shown in red.

**(a) DNA sequence**

GCTCTTCTATGTCTATTAACAAAATTACTGATAACTTAACAACTTCTGCTGAACACAGTGAAAACCACCACGACAACAACCAAGTAACTGCTGGTAAAAACATGTCTTCTCGTTCATTATTATACGTTGCTGTAGCTTTAGGTTTATTCACTTGTGTAGCTGCTCAAGCTGAAACACAAGCTGCTTTAACACAAGAATTAAAAGACGAAGAAGGTTTATCTTTAGAACGTTCACAATTCTCTGTTTCTTTAGAATCATCTTCTACTACAAAAGGTACTGCTACTCCAAGTGGTGCAGAATCTACTGCTTTAACAACAGGTCTTCAATATAACGAATTAGCAAGTGAACTTAAACAATTACACCAAGGTGCTTCTCTTTCACGTTTATGATCACAAAAAAAACCTGCTTCTGTAGATTCTGTTAACTCTGTTGCTTCAATGAACAAAGGTGATGCTGCAGGTTCTAATTTAGCAACTAAAGCTTCTGTATTATCTGATCCAGCTGTAAAAGCATCTCCAGCTAACTTACGTACAGTAAACACTTTAGACCAAGGTTTATTAGGTATGACACGTGAACAAAAAATGGCTATTAAATCAGCTGAAAACTCAGCTTCAGTACAAGCTTTACAATCTTCAGGTTTATTCCACTCTTTCAACATTTACGATGCTAACACTCACTTATTAGAAGATTTCGATGGTGACTCATTCTACAGTACATTCTCAGTAACATTCGATGCTGATGTTGATGGTATTGGTTACAACGAATACGCTGATGTTTATGCTGAATTATACGTTTCTCAAGAAGGTGGTCCATGATTACACTACTACTCAACTGAAGTATTCTCAATTGCTGGTAACTCATCTTACGATGATTACCGTGTTTTAACAACTTTACAATCAGGTTACCAAACAGCTCACTACGACGTTTTAATTGATTTATACGAAGTTGGTGTATCAAACCCAGTAGCTACTTTATCTTCAAACGATACTAACGCTTTATACGCTTTACCATTAGAATCTCGTGACCGTGACCCTATTTACGTTGAACCACACGTAGATACTTACATTGAAGTTGAAGCTGGTGGTGCTTTATCTTGGTGGGAATTATTAGTATTAGTTTCTTTAGGTTTATTAGCTATTCGTAAATCACCACAATGTTACCCATACGATGTTCCAGATTATGCTTAATAAGCATGC

**(b) Translated sequence**

MSINKITDNLTTSAEHSENHHDNNQVTAGKNMSSRSLLYVAVALGLFTCVAAQAETQAALTQELKDEEGLSLERSQFSVSLESSSTTKGTATPSGAESTALTTGLQYNELASELKQLHQGASLSRLWSQKKPASVDSVNSVASMNKGDAAGSNLATKASVLSDPAVKASPANLRTVNTLDQGLLGMTREQKMAIKSAENSASVQALQSSGLFHSFNIYDANTHLLEDFDGDSFYSTFSVTFDADVDGIGYNEYADVYAELYVSQEGGPWLHYYSTEVFSIAGNSSYDDYRVLTTLQSGYQTAHYDVLIDLYEVGVSNPVATLSSNDTNALYALPLESRDRDPIYVEPHVDTYIEVEAGGALSWWELLVLVSLGLLAIRKSPQCYPYDVPDYA

**5. Plasmid pPsaA***

*PsaA* exon 3 (forwards orientation) is highlighted in turquoise. The TGG🡺TGA codon alteration is shown in green.

CCCATCAAGCTTATCGATACCGTCGACCTCGAGGGGGGGCCCGGTACCCAATTCGCCCTATAGTGAGTCGTATTACAATTCACTGGCCGTCGTTTTACAACGTCGTGACTGGGAAAACCCTGGCGTTACCCAACTTAATCGCCTTGCAGCACATCCCCCTTTCGCCAGCTGGCGTAATAGCGAAGAGGCCCGCACCGATCGCCCTTCCCAACAGTTGCGCAGCCTGAATGGCGAATGGAAATTGTAAGCGTTAATATTTTGTTAAAATTCGCGTTAAATTTTTGTTAAATCAGCTCATTTTTTAACCAATAGGCCGAAATCGGCAAAATCCCTTATAAATCAAAAGAATAGACCGAGATAGGGTTGAGTGTTGTTCCAGTTTGGAACAAGAGTCCACTATTAAAGAACGTGGACTCCAACGTCAAAGGGCGAAAAACCGTCTATCAGGGCGATGGCCCACTACGTGAACCATCACCCTAATCAAGTTTTTTGGGGTCGAGGTGCCGTAAAGCACTAAATCGGAACCCTAAAGGGAGCCCCCGATTTAGAGCTTGACGGGGAAAGCCGGCGAACGTGGCGAGAAAGGAAGGGAAGAAAGCGAAAGGAGCGGGCGCTAGGGCGCTGGCAAGTGTAGCGGTCACGCTGCGCGTAACCACCACACCCGCCGCGCTTAATGCGCCGCTACAGGGCGCGTCAGGTGGCACTTTTCGGGGAAATGTGCGCGGAACCCCTATTTGTTTATTTTTCTAAATACATTCAAATATGTATCCGCTCATGAGACAATAACCCTGATAAATGCTTCAATAATATTGAAAAAGGAAGAGTATGAGTATTCAACATTTCCGTGTCGCCCTTATTCCCTTTTTTGCGGCATTTTGCCTTCCTGTTTTTGCTCACCCAGAAACGCTGGTGAAAGTAAAAGATGCTGAAGATCAGTTGGGTGCACGAGTGGGTTACATCGAACTGGATCTCAACAGCGGTAAGATCCTTGAGAGTTTTCGCCCCGAAGAACGTTTTCCAATGATGAGCACTTTTAAAGTTCTGCTATGTGGCGCGGTATTATCCCGTATTGACGCCGGGCAAGAGCAACTCGGTCGCCGCATACACTATTCTCAGAATGACTTGGTTGAGTACTCACCAGTCACAGAAAAGCATCTTACGGATGGCATGACAGTAAGAGAATTATGCAGTGCTGCCATAACCATGAGTGATAACACTGCGGCCAACTTACTTCTGACAACGATCGGAGGACCGAAGGAGCTAACCGCTTTTTTGCACAACATGGGGGATCATGTAACTCGCCTTGATCGTTGGGAACCGGAGCTGAATGAAGCCATACCAAACGACGAGCGTGACACCACGATGCCTGTAGCAATGGCAACAACGTTGCGCAAACTATTAACTGGCGAACTACTTACTCTAGCTTCCCGGCAACAATTAATAGACTGGATGGAGGCGGATAAAGTTGCAGGACCACTTCTGCGCTCGGCCCTTCCGGCTGGCTGGTTTATTGCTGATAAATCTGGAGCCGGTGAGCGTGGGTCTCGCGGTATCATTGCAGCACTGGGGCCAGATGGTAAGCCCTCCCGTATCGTAGTTATCTACACGACGGGGAGTCAGGCAACTATGGATGAACGAAATAGACAGATCGCTGAGATAGGTGCCTCACTGATTAAGCATTGGTAACTGTCAGACCAAGTTTACTCATATATACTTTAGATTGATTTAAAACTTCATTTTTAATTTAAAAGGATCTAGGTGAAGATCCTTTTTGATAATCTCATGACCAAAATCCCTTAACGTGAGTTTTCGTTCCACTGAGCGTCAGACCCCGTAGAAAAGATCAAAGGATCTTCTTGAGATCCTTTTTTTCTGCGCGTAATCTGCTGCTTGCAAACAAAAAAACCACCGCTACCAGCGGTGGTTTGTTTGCCGGATCAAGAGCTACCAACTCTTTTTCCGAAGGTAACTGGCTTCAGCAGAGCGCAGATACCAAATACTGTCCTTCTAGTGTAGCCGTAGTTAGGCCACCACTTCAAGAACTCTGTAGCACCGCCTACATACCTCGCTCTGCTAATCCTGTTACCAGTGGCTGCTGCCAGTGGCGATAAGTCGTGTCTTACCGGGTTGGACTCAAGACGATAGTTACCGGATAAGGCGCAGCGGTCGGGCTGAACGGGGGGTTCGTGCACACAGCCCAGCTTGGAGCGAACGACCTACACCGAACTGAGATACCTACAGCGTGAGCTATGAGAAAGCGCCACGCTTCCCGAAGGGAGAAAGGCGGACAGGTATCCGGTAAGCGGCAGGGTCGGAACAGGAGAGCGCACGAGGGAGCTTCCAGGGGGAAACGCCTGGTATCTTTATAGTCCTGTCGGGTTTCGCCACCTCTGACTTGAGCGTCGATTTTTGTGATGCTCGTCAGGGGGGCGGAGCCTATGGAAAAACGCCAGCAACGCGGCCTTTTTACGGTTCCTGGCCTTTTGCTGGCCTTTTGCTCACATGTTCTTTCCTGCGTTATCCCCTGATTCTGTGGATAACCGTATTACCGCCTTTGAGTGAGCTGATACCGCTCGCCGCAGCCGAACGACCGAGCGCAGCGAGTCAGTGAGCGAGGAAGCGGAAGAGCGCCCAATACGCAAACCGCCTCTCCCCGCGCGTTGGCCGATTCATTAATGCAGCTGGCACGACAGGTTTCCCGACTGGAAAGCGGGCAGTGAGCGCAACGCAATTAATGTGAGTTAGCTCACTCATTAGGCACCCCAGGCTTTACACTTTATGCTTCCGGCTCGTATGTTGTGTGGAATTGTGAGCGGATAACAATTTCACACAGGAAACAGCTATGACCATGATTACGCCAAGCTCGAAATTAACCCTCACTAAAGGGAACAAAAGCTGGAGCTCCACCGCGGTGGCGGCCGCTctaggtatatacattcaccctttaaggctacccggcagttagttacggcttacgttccataaaatattggcatattttataaattattttatagatcatatattttgtaaatatataatattacgcataacacatttatttaaaaacatataatattacgcataacacatttatttaaaaacagcaaaaacttgcgtcaaatccctataggatattttttatttatggcacgagcaggtttacacgctccgtcaggacgccggcacgtagttggaaagtatgtcccccttgcccggaaggggaaaggaggagacaaatttatttattgtatataaatagcatgactttccaagcgagttaacataaacaaactgcgcgagttaacataaacaaacttcctctctggggaggcatggcagcaaatggcaccctaaatacatgcgcttttaacacagataattataaacaagcatagcgttaaatctgctaccttggattaaatcattagaaagaatttgagccgtgtgcagtgaaaattgcatgcacggctcttaaggtttaaataaatttttaaagaagaaaatttaactcctaactatgtacttccatggtgcacgtttttcaaactatgaagcttggttaagtgaccctactcacattaaaccaagtgctcaagtagtatggcctattgtaggtcaagaaattttaaacggtgatgtaggtggtggtttccaaggtattcaaattacttctggtttcttccaattatggcgtgctagtggtattactagtgaattacaactttatactacagcaattggtggtttagtaatggctgctgcaatgttctttgctggttggttccactaccacaaagctgctccaaaactagaatggttccaaaacgttgaatcaatgttaaaccaccacttaggtggtcttcttggtttaggtagtttagcttgggctggtcaccaaattcacgtttctttaccagtaaacaaattattagatgctggtgtagatccaaaagaaattccacttcctcatgatttattattaaatcgtgctattatggctgacttatacccaagttttgctaaaggtattgctcctttctttactttaaactggagtgaatacagtgatttcttaacatttaaaggtggtttaaaccctgttactggtggtctttggttaagtgatactgctcaccaccacgtagctattgctgtattattcttagtagctggtcacatgtatcgtactaactggggtattggtcacagtatgaaagaaattttagaagctcaccgtggtccatttacaggtgaaggtcacgttggtttatatgaaattttaacaacttcttggcatgcacaattagctattaacttagctttatttggttcgttatcaattattgtagctcaccacatgtacgcaatgcctccatacccttatttagctactgattacggtacacaattatcattatttacacaccacacatggattggtggtttctgtattgttggtgctggtgctcacgcagctattttcatggttcgtgactacgatcctactaataactacaacaacttattagaccgtgtaattcgtcaccgtgatgctattatttctcacttaaactgggtttgtattttcttaggtttccacagctttggtttatacatccacaacgatacaatgagtgctttaggtcgtcctcaagacatgttctcagatactgctatccaacttcaaccagtatttgctcaatggattcaaaatacacacttcttagctccacaattaacagcaccaaatgctttagctgctacaagtttaacttggggtggtgagctaggtgctcatggcggtaaagtagctatgatgcctatttctttaggtacttctgactttatggttcaccacattcacgctttcacaattcacgtaactgtgttaattcttctgaaaggtgttttatttgctcgtagctctcgtcttatcccagataaagctaacttaggtttccgtttcccttgtgacggtcctggtcgtggcggtacttgtcaggtttctgcttgggaccacgtattcttaggtcttttctggatgtacaacagcttatcaattgtaattttccacttcagctggaagatgcaatctgatgtttggggtacggttacagcttctggtgtttctcacattactggtggtaactttgcacaaagcgctaacacaatcaacggttggttacgtgacttcttatgggcacaatcatcacaagtaatccaatcatacggttcagctctatctgcttatggtttaattttcttaggtgctcacttcgtatgggcattctcgttaatgttcttattctctggtcgtggttactgAcaagaacttatcgaatcaattgtatgggctcacaacaaacttaaagttgcacctgcaattcaaccacgtgctttaagtattactcaaggtcgtgctgttggtgtagctcactaccttttaggtggtattgctactacatggtcgttcttcttagcacgtatcatttctgtaggttaacatttaatactttttaatacatatatgcctaagtttatctttaaagataaacttagccatatgtgttaagttatctaacaaggttacctttttatttctctttagatatataaacattaaaaactaccgtgatcgttacactttagataactggaagggggaaaaatcatgtattcgctggaaggcgcacctcctactgcctactgcgcagcattaaaatgctgtagatattggtatcttacaaaggacagtagtacacaattaaacgcgttggaatatttatattttcattagagAATTgggtaccgagctccaccgcggtggcggccgctctagctagaactagtggatcgcactctaccgattgagttacatccgctttagtatgttactatttcttttattataacttataaaatataatacataaagataaattctataataaaaagctaagattttatttttctggcacatcgtaatttataaagacaggcaaatttaaacaaaagataactttagaacttaattttaaaaatgtaaaatgatgtttaggtatttaacctaaacaccataaaaataaaaacgatgtttatgctattcacataaacatcatgaaaaataaaaattaaagtttgtcaatagtatcaaattcgaatttaatttctttccaaacttcacatgcagcagcaagttctggagaccatttacaagctgaacgaattacgtcgccaccttcacgagcaaggtcacgaccttcgttacgagcttgagtacaagcttgcatgcctgcagTTATTTGCCAACTACCTTAGTGATCTCGCCTTTCACGTAGTGGACAAATTCTTCCAACTGATCTGCGCGCGAGGCCAAGCGATCTTCTTCTTGTCCAAGATAAGCCTGTCTAGCTTCAAGTATGACGGGCTGATACTGGGCCGGCAGGCGCTCCATTGCCCAGTCGGCAGCGACATCCTTCGGCGCGATTTTGCCGGTTACTGCGCTGTACCAAATGCGGGACAACGTAAGCACTACATTTCGCTCATCGCCAGCCCAGTCGGGCGGCGAGTTCCATAGCGTTAAGGTTTCATTTAGCGCCTCAAATAGATCCTGTTCAGGAACCGGATCAAAGAGTTCCTCCGCCGCTGGACCTACCAAGGCAACGCTATGTTCTCTTGCTTTTGTCAGCAAGATAGCCAGATCAATGTCGATCGTGGCTGGCTCGAAGATACCTGCAAGAATGTCATTGCGCTGCCATTCTCCAAATTGCAGTTCGCGCTTAGCTGGATAACGCCACGGAATGATGTCGTCGTGCACAACAATGGTGACTTCTACAGCGCGGAGAATCTCGCTCTCTCCAGGGGAAGCCGAAGTTTCCAAAAGGTCGTTGATCAAAGCTCGCCGCGTTGTTTCATCAAGCCTTACGGTCACCGTAACCAGCAAATCAATATCACTGTGTGGCTTCAGGCCGCCATCCACTGCGGAGCCGTACAAATGTACGGCCAGCAACGTCGGTTCGAGATGGCGCTCGATGACGCCAACTACCTCTGATAGTTGAGTTGATACTTCGGCGATAACCGCTTCACGAGCCATggacattttcacttctggagtgtattgttcaattaaatctttaataagattactaagttcttctggagtacgcattgccataaaaaagaaaaaataaataaaagattaaaaaagtttatttttaaaatctttctcgagaattttaaataagtttaaaattcaacaaaaatagtgagtggtaagatcacttgttaacaaaagtaatggttcacccttgtcatatttaaatactaaaattcatttgcccgaagaggacaaatttatttattgcattaaaatccctaagtttacttgcccgtaaggggaagggggggacgtccacaggcgtcgtaagcaactaaagtttatgacgccgattgctttgttaggaaaatataaatatcccataagaaaaggtcctttaaaggttttatggactaaataaaaaagatagcataagcattaaaatcatgcaaattaaaaaaaaggtaaatgtatttataaaaaggtaaatgtatttatatagtatttatattatagcataataataaatatatttataaattgattgttcttagagctaaaagagaagaacaatgggtttataggtattttgagatccagttataaaaatgacttttgacgtttatggtatataaacactgcctctaataaagtcatcgataagcttgatcccatatagccaatggcttaaggagtgtcataggaataactagtcatgcacattttcctaaaaatctaaaatgttattagaagctatacaaaaattaaaaattatgttattatatatttattaaaatttagacttatcacggggatatggcggaatggtagacgctacggacttaaaatccgttcttgtgcgaacaaggtgagggttcaagtccctctttccccatttataaataaaaatcaagtcaatattttaacattgtaaactaaaaaataagtgtaaaataactagggtaaatagttaagaattggtagtttttgaactaaacagtgacataaaactggttaaaagacatccctgtaagagaaatgcatatggtgaattacacaataaatttaaaaataaagctctgacaagcctcttcccctttgcgatatacatgctctgttagatataattctgcctaagttcctaacaaagggtttactttttctcggggaatggttttgctactttgttttaaatctttttcaaagaatgtcaatctcctataaagggaggaacattactcatggtctcacaattttaataaactagtttcttataatctattttataaaaataaatctaaatatatttcttatgaattttgttaatttagaacaaattgaaaattctttacgtaatgctactttttgcatgctttttttaacaacatttttatattggttttatactgcattttacagtacaaatcctcaacaaattataaatccattgtcattaacaaacattaaaactaattttacttatcctgacgggtttcaagcaaacaatattaatgtttctacaacttatttaccgattaaccctgtattaaataccgaaagagaagagcaacccgaagctaacggaacgaatgggctgttgggtgtttcttcattagtagtaaatttaaaatcaatagcaatcccacgcattatgatgggtgtttctaatttattattagtattattattacttgttcgttgggaaaaatcaggtcatttcccattaagtaatttatatgaatctttaatgtttttagcgtggtgttgtacatttttatatttattatattgtacaagttttactttattggttgaaaaaatgttaggttcattaattgcaccttgtagtttattaatgaatgcatttgctacttttagtttaccaaaagaaatgcaacaagcatcaccattagtaccagctttacaatcaaattggttaatgatgcatgttactgtaatgattattagttatgccacattaattattggatcgttattgtcgattttatttttgattttatttaaacacaaaaaaggtacacccaaaaagtatgataactttattaacaatttagatgcattaagttatcgcattattggattaggttttccttttttaactattgggattttatctggggctgtgtgggctaatgaagcatggggatcatattggagttgggatcc

**6. Plasmid pWUCA1**

The *trnW_UCA_* region is colour coded as in section 2 above. *PsaA* exon 3 (forwards orientation) is highlighted in turquoise. The *aadA* spectinomycin resistance gene (reverse orientation) is highlighted in pink and its promoter/UTRs in green.

CCCATCAAGCTTATCGATACCGTCGACCTCGAGGGGGGGCCCGGTACCCAATTCGCCCTATAGTGAGTCGTATTACAATTCACTGGCCGTCGTTTTACAACGTCGTGACTGGGAAAACCCTGGCGTTACCCAACTTAATCGCCTTGCAGCACATCCCCCTTTCGCCAGCTGGCGTAATAGCGAAGAGGCCCGCACCGATCGCCCTTCCCAACAGTTGCGCAGCCTGAATGGCGAATGGAAATTGTAAGCGTTAATATTTTGTTAAAATTCGCGTTAAATTTTTGTTAAATCAGCTCATTTTTTAACCAATAGGCCGAAATCGGCAAAATCCCTTATAAATCAAAAGAATAGACCGAGATAGGGTTGAGTGTTGTTCCAGTTTGGAACAAGAGTCCACTATTAAAGAACGTGGACTCCAACGTCAAAGGGCGAAAAACCGTCTATCAGGGCGATGGCCCACTACGTGAACCATCACCCTAATCAAGTTTTTTGGGGTCGAGGTGCCGTAAAGCACTAAATCGGAACCCTAAAGGGAGCCCCCGATTTAGAGCTTGACGGGGAAAGCCGGCGAACGTGGCGAGAAAGGAAGGGAAGAAAGCGAAAGGAGCGGGCGCTAGGGCGCTGGCAAGTGTAGCGGTCACGCTGCGCGTAACCACCACACCCGCCGCGCTTAATGCGCCGCTACAGGGCGCGTCAGGTGGCACTTTTCGGGGAAATGTGCGCGGAACCCCTATTTGTTTATTTTTCTAAATACATTCAAATATGTATCCGCTCATGAGACAATAACCCTGATAAATGCTTCAATAATATTGAAAAAGGAAGAGTATGAGTATTCAACATTTCCGTGTCGCCCTTATTCCCTTTTTTGCGGCATTTTGCCTTCCTGTTTTTGCTCACCCAGAAACGCTGGTGAAAGTAAAAGATGCTGAAGATCAGTTGGGTGCACGAGTGGGTTACATCGAACTGGATCTCAACAGCGGTAAGATCCTTGAGAGTTTTCGCCCCGAAGAACGTTTTCCAATGATGAGCACTTTTAAAGTTCTGCTATGTGGCGCGGTATTATCCCGTATTGACGCCGGGCAAGAGCAACTCGGTCGCCGCATACACTATTCTCAGAATGACTTGGTTGAGTACTCACCAGTCACAGAAAAGCATCTTACGGATGGCATGACAGTAAGAGAATTATGCAGTGCTGCCATAACCATGAGTGATAACACTGCGGCCAACTTACTTCTGACAACGATCGGAGGACCGAAGGAGCTAACCGCTTTTTTGCACAACATGGGGGATCATGTAACTCGCCTTGATCGTTGGGAACCGGAGCTGAATGAAGCCATACCAAACGACGAGCGTGACACCACGATGCCTGTAGCAATGGCAACAACGTTGCGCAAACTATTAACTGGCGAACTACTTACTCTAGCTTCCCGGCAACAATTAATAGACTGGATGGAGGCGGATAAAGTTGCAGGACCACTTCTGCGCTCGGCCCTTCCGGCTGGCTGGTTTATTGCTGATAAATCTGGAGCCGGTGAGCGTGGGTCTCGCGGTATCATTGCAGCACTGGGGCCAGATGGTAAGCCCTCCCGTATCGTAGTTATCTACACGACGGGGAGTCAGGCAACTATGGATGAACGAAATAGACAGATCGCTGAGATAGGTGCCTCACTGATTAAGCATTGGTAACTGTCAGACCAAGTTTACTCATATATACTTTAGATTGATTTAAAACTTCATTTTTAATTTAAAAGGATCTAGGTGAAGATCCTTTTTGATAATCTCATGACCAAAATCCCTTAACGTGAGTTTTCGTTCCACTGAGCGTCAGACCCCGTAGAAAAGATCAAAGGATCTTCTTGAGATCCTTTTTTTCTGCGCGTAATCTGCTGCTTGCAAACAAAAAAACCACCGCTACCAGCGGTGGTTTGTTTGCCGGATCAAGAGCTACCAACTCTTTTTCCGAAGGTAACTGGCTTCAGCAGAGCGCAGATACCAAATACTGTCCTTCTAGTGTAGCCGTAGTTAGGCCACCACTTCAAGAACTCTGTAGCACCGCCTACATACCTCGCTCTGCTAATCCTGTTACCAGTGGCTGCTGCCAGTGGCGATAAGTCGTGTCTTACCGGGTTGGACTCAAGACGATAGTTACCGGATAAGGCGCAGCGGTCGGGCTGAACGGGGGGTTCGTGCACACAGCCCAGCTTGGAGCGAACGACCTACACCGAACTGAGATACCTACAGCGTGAGCTATGAGAAAGCGCCACGCTTCCCGAAGGGAGAAAGGCGGACAGGTATCCGGTAAGCGGCAGGGTCGGAACAGGAGAGCGCACGAGGGAGCTTCCAGGGGGAAACGCCTGGTATCTTTATAGTCCTGTCGGGTTTCGCCACCTCTGACTTGAGCGTCGATTTTTGTGATGCTCGTCAGGGGGGCGGAGCCTATGGAAAAACGCCAGCAACGCGGCCTTTTTACGGTTCCTGGCCTTTTGCTGGCCTTTTGCTCACATGTTCTTTCCTGCGTTATCCCCTGATTCTGTGGATAACCGTATTACCGCCTTTGAGTGAGCTGATACCGCTCGCCGCAGCCGAACGACCGAGCGCAGCGAGTCAGTGAGCGAGGAAGCGGAAGAGCGCCCAATACGCAAACCGCCTCTCCCCGCGCGTTGGCCGATTCATTAATGCAGCTGGCACGACAGGTTTCCCGACTGGAAAGCGGGCAGTGAGCGCAACGCAATTAATGTGAGTTAGCTCACTCATTAGGCACCCCAGGCTTTACACTTTATGCTTCCGGCTCGTATGTTGTGTGGAATTGTGAGCGGATAACAATTTCACACAGGAAACAGCTATGACCATGATTACGCCAAGCTCGAAATTAACCCTCACTAAAGGGAACAAAAGCTGGAGCTCCACCGCGGTGGCGGCCGCTctaggtatatacattcaccctttaaggctacccggcagttagttacggcttacgttccataaaatattggcatattttataaattattttatagatcatatattttgtaaatatataatattacgcataacacatttatttaaaaacatataatattacgcataacacatttatttaaaaacagcaaaaacttgcgtcaaatccctataggatattttttatttatggcacgagcaggtttacacgctccgtcaggacgccggcacgtagttggaaagtatgtcccccttgcccggaaggggaaaggaggagacaaatttatttattgtatataaatagcatgactttccaagcgagttaacataaacaaactgcgcgagttaacataaacaaacttcctctctggggaggcatggcagcaaatggcaccctaaatacatgcgcttttaacacagataattataaacaagcatagcgttaaatctgctaccttggattaaatcattagaaagaatttgagccgtgtgcagtgaaaattgcatgcacggctcttaaggtttaaataaatttttaaagaagaaaatttaactcctaactatgtacttccatggtgcacgtttttcaaactatgaagcttggttaagtgaccctactcacattaaaccaagtgctcaagtagtatggcctattgtaggtcaagaaattttaaacggtgatgtaggtggtggtttccaaggtattcaaattacttctggtttcttccaattatggcgtgctagtggtattactagtgaattacaactttatactacagcaattggtggtttagtaatggctgctgcaatgttctttgctggttggttccactaccacaaagctgctccaaaactagaatggttccaaaacgttgaatcaatgttaaaccaccacttaggtggtcttcttggtttaggtagtttagcttgggctggtcaccaaattcacgtttctttaccagtaaacaaattattagatgctggtgtagatccaaaagaaattccacttcctcatgatttattattaaatcgtgctattatggctgacttatacccaagttttgctaaaggtattgctcctttctttactttaaactggagtgaatacagtgatttcttaacatttaaaggtggtttaaaccctgttactggtggtctttggttaagtgatactgctcaccaccacgtagctattgctgtattattcttagtagctggtcacatgtatcgtactaactggggtattggtcacagtatgaaagaaattttagaagctcaccgtggtccatttacaggtgaaggtcacgttggtttatatgaaattttaacaacttcttggcatgcacaattagctattaacttagctttatttggttcgttatcaattattgtagctcaccacatgtacgcaatgcctccatacccttatttagctactgattacggtacacaattatcattatttacacaccacacatggattggtggtttctgtattgttggtgctggtgctcacgcagctattttcatggttcgtgactacgatcctactaataactacaacaacttattagaccgtgtaattcgtcaccgtgatgctattatttctcacttaaactgggtttgtattttcttaggtttccacagctttggtttatacatccacaacgatacaatgagtgctttaggtcgtcctcaagacatgttctcagatactgctatccaacttcaaccagtatttgctcaatggattcaaaatacacacttcttagctccacaattaacagcaccaaatgctttagctgctacaagtttaacttggggtggtgagctaggtgctcatggcggtaaagtagctatgatgcctatttctttaggtacttctgactttatggttcaccacattcacgctttcacaattcacgtaactgtgttaattcttctgaaaggtgttttatttgctcgtagctctcgtcttatcccagataaagctaacttaggtttccgtttcccttgtgacggtcctggtcgtggcggtacttgtcaggtttctgcttgggaccacgtattcttaggtcttttctggatgtacaacagcttatcaattgtaattttccacttcagctggaagatgcaatctgatgtttggggtacggttacagcttctggtgtttctcacattactggtggtaactttgcacaaagcgctaacacaatcaacggttggttacgtgacttcttatgggcacaatcatcacaagtaatccaatcatacggttcagctctatctgcttatggtttaattttcttaggtgctcacttcgtatgggcattctcgttaatgttcttattctctggtcgtggttactggcaagaacttatcgaatcaattgtatgggctcacaacaaacttaaagttgcacctgcaattcaaccacgtgctttaagtattactcaaggtcgtgctgttggtgtagctcactaccttttaggtggtattgctactacatggtcgttcttcttagcacgtatcatttctgtaggttaacatttaatactttttaatacatatatgcctaagtttatctttaaagataaacttagccatatgtgttaagttatctaacaaggttacctttttatttctctttagatatataaacattaaaaactaccgtgatcgttacactttagataactggaagggggaaaaatcatgtattcgctggaaggcgcacctcctactgcctactgcgcagcattaaaatgctgtagatattggtatcttacaaaggacagtagtacacaattaa**ACGCGT**taacccatgattaacaactatatcaataaaatcaatttgtagtgaaatactctga**ttgaca**ttaaaataataccatgataaaaat**tataat**aacaaattttacgtccttagttcagtcggtagaacgcaggttt**tca**aaacctgatgtcgtgggttcaattcctacagggcgtgtttttcctaatgtactttgttgtaaaagtggctggtttaacctttttaggtttcggattgaacaataatggcagttaagagtcactaaagctgctgtatag**ACGCGT**tggaatatttatattttcattagagAATTgggtaccgagctccaccgcggtggcggccgctctagctagaactagtggatcgcactctaccgattgagttacatccgctttagtatgttactatttcttttattataacttataaaatataatacataaagataaattctataataaaaagctaagattttatttttctggcacatcgtaatttataaagacaggcaaatttaaacaaaagataactttagaacttaattttaaaaatgtaaaatgatgtttaggtatttaacctaaacaccataaaaataaaaacgatgtttatgctattcacataaacatcatgaaaaataaaaattaaagtttgtcaatagtatcaaattcgaatttaatttctttccaaacttcacatgcagcagcaagttctggagaccatttacaagctgaacgaattacgtcgccaccttcacgagcaaggtcacgaccttcgttacgagcttgagtacaagcttgcatgcctgcagTTATTTGCCAACTACCTTAGTGATCTCGCCTTTCACGTAGTGGACAAATTCTTCCAACTGATCTGCGCGCGAGGCCAAGCGATCTTCTTCTTGTCCAAGATAAGCCTGTCTAGCTTCAAGTATGACGGGCTGATACTGGGCCGGCAGGCGCTCCATTGCCCAGTCGGCAGCGACATCCTTCGGCGCGATTTTGCCGGTTACTGCGCTGTACCAAATGCGGGACAACGTAAGCACTACATTTCGCTCATCGCCAGCCCAGTCGGGCGGCGAGTTCCATAGCGTTAAGGTTTCATTTAGCGCCTCAAATAGATCCTGTTCAGGAACCGGATCAAAGAGTTCCTCCGCCGCTGGACCTACCAAGGCAACGCTATGTTCTCTTGCTTTTGTCAGCAAGATAGCCAGATCAATGTCGATCGTGGCTGGCTCGAAGATACCTGCAAGAATGTCATTGCGCTGCCATTCTCCAAATTGCAGTTCGCGCTTAGCTGGATAACGCCACGGAATGATGTCGTCGTGCACAACAATGGTGACTTCTACAGCGCGGAGAATCTCGCTCTCTCCAGGGGAAGCCGAAGTTTCCAAAAGGTCGTTGATCAAAGCTCGCCGCGTTGTTTCATCAAGCCTTACGGTCACCGTAACCAGCAAATCAATATCACTGTGTGGCTTCAGGCCGCCATCCACTGCGGAGCCGTACAAATGTACGGCCAGCAACGTCGGTTCGAGATGGCGCTCGATGACGCCAACTACCTCTGATAGTTGAGTTGATACTTCGGCGATAACCGCTTCACGAGCCATggacattttcacttctggagtgtattgttcaattaaatctttaataagattactaagttcttctggagtacgcattgccataaaaaagaaaaaataaataaaagattaaaaaagtttatttttaaaatctttctcgagaattttaaataagtttaaaattcaacaaaaatagtgagtggtaagatcacttgttaacaaaagtaatggttcacccttgtcatatttaaatactaaaattcatttgcccgaagaggacaaatttatttattgcattaaaatccctaagtttacttgcccgtaaggggaagggggggacgtccacaggcgtcgtaagcaactaaagtttatgacgccgattgctttgttaggaaaatataaatatcccataagaaaaggtcctttaaaggttttatggactaaataaaaaagatagcataagcattaaaatcatgcaaattaaaaaaaaggtaaatgtatttataaaaaggtaaatgtatttatatagtatttatattatagcataataataaatatatttataaattgattgttcttagagctaaaagagaagaacaatgggtttataggtattttgagatccagttataaaaatgacttttgacgtttatggtatataaacactgcctctaataaagtcatcgataagcttgatcccatatagccaatggcttaaggagtgtcataggaataactagtcatgcacattttcctaaaaatctaaaatgttattagaagctatacaaaaattaaaaattatgttattatatatttattaaaatttagacttatcacggggatatggcggaatggtagacgctacggacttaaaatccgttcttgtgcgaacaaggtgagggttcaagtccctctttccccatttataaataaaaatcaagtcaatattttaacattgtaaactaaaaaataagtgtaaaataactagggtaaatagttaagaattggtagtttttgaactaaacagtgacataaaactggttaaaagacatccctgtaagagaaatgcatatggtgaattacacaataaatttaaaaataaagctctgacaagcctcttcccctttgcgatatacatgctctgttagatataattctgcctaagttcctaacaaagggtttactttttctcggggaatggttttgctactttgttttaaatctttttcaaagaatgtcaatctcctataaagggaggaacattactcatggtctcacaattttaataaactagtttcttataatctattttataaaaataaatctaaatatatttcttatgaattttgttaatttagaacaaattgaaaattctttacgtaatgctactttttgcatgctttttttaacaacatttttatattggttttatactgcattttacagtacaaatcctcaacaaattataaatccattgtcattaacaaacattaaaactaattttacttatcctgacgggtttcaagcaaacaatattaatgtttctacaacttatttaccgattaaccctgtattaaataccgaaagagaagagcaacccgaagctaacggaacgaatgggctgttgggtgtttcttcattagtagtaaatttaaaatcaatagcaatcccacgcattatgatgggtgtttctaatttattattagtattattattacttgttcgttgggaaaaatcaggtcatttcccattaagtaatttatatgaatctttaatgtttttagcgtggtgttgtacatttttatatttattatattgtacaagttttactttattggttgaaaaaatgttaggttcattaattgcaccttgtagtttattaatgaatgcatttgctacttttagtttaccaaaagaaatgcaacaagcatcaccattagtaccagctttacaatcaaattggttaatgatgcatgttactgtaatgattattagttatgccacattaattattggatcgttattgtcgattttatttttgattttatttaaacacaaaaaaggtacacccaaaaagtatgataactttattaacaatttagatgcattaagttatcgcattattggattaggttttccttttttaactattgggattttatctggggctgtgtgggctaatgaagcatggggatcatattggagttgggatcc

**7. Plasmid pWUCA2**

The *trnW_UCA_* region is colour coded as in section 2 above. The *psaA* exon 1 promoter and 5’ UTR are highlighted in pink and the SapI and SphI sites for inserting a gene of interest are highlighted in yellow. The *psbH* gene (reverse orientation) is highlighted in turquoise.

AGCTTGGCACTGGCCGTCGTTTTACAACGTCGTGACTGGGAAAACCCTGGCGTTACCCAACTTAATCGCCTTGCAGCACATCCCCCTTTCGCCAGCTGGCGTAATAGCGAAGAGGCCCGCACCGATCGCCCTTCCCAACAGTTGCGCAGCCTGAATGGCGAATGGCGCCTGATGCGGTATTTTCTCCTTACGCATCTGTGCGGTATTTCACACCGCATATGGTGCACTCTCAGTACAATCTGCTCTGATGCCGCATAGTTAAGCCAGCCCCGACACCCGCCAACACCCGCTGACGCGCCCTGACGGGCTTGTCTGCTCCCGGCATCCGCTTACAGACAAGCTGTGACCGTCTCCGGGAGCTGCATGTGTCAGAGGTTTTCACCGTCATCACCGAAACGCGCGAGACGAAAGGGCCTCGTGATACGCCTATTTTTATAGGTTAATGTCATGATAATAATGGTTTCTTAGACGTCAGGTGGCACTTTTCGGGGAAATGTGCGCGGAACCCCTATTTGTTTATTTTTCTAAATACATTCAAATATGTATCCGCTCATGAGACAATAACCCTGATAAATGCTTCAATAATATTGAAAAAGGAAGAGTATGAGTATTCAACATTTCCGTGTCGCCCTTATTCCCTTTTTTGCGGCATTTTGCCTTCCTGTTTTTGCTCACCCAGAAACGCTGGTGAAAGTAAAAGATGCTGAAGATCAGTTGGGTGCACGAGTGGGTTACATCGAACTGGATCTCAACAGCGGTAAGATCCTTGAGAGTTTTCGCCCCGAAGAACGTTTTCCAATGATGAGCACTTTTAAAGTTCTGCTATGTGGCGCGGTATTATCCCGTATTGACGCCGGGCAAGAGCAACTCGGTCGCCGCATACACTATTCTCAGAATGACTTGGTTGAGTACTCACCAGTCACAGAAAAGCATCTTACGGATGGCATGACAGTAAGAGAATTATGCAGTGCTGCCATAACCATGAGTGATAACACTGCGGCCAACTTACTTCTGACAACGATCGGAGGACCGAAGGAGCTAACCGCTTTTTTGCACAACATGGGGGATCATGTAACTCGCCTTGATCGTTGGGAACCGGAGCTGAATGAAGCCATACCAAACGACGAGCGTGACACCACGATGCCTGTAGCAATGGCAACAACGTTGCGCAAACTATTAACTGGCGAACTACTTACTCTAGCTTCCCGGCAACAATTAATAGACTGGATGGAGGCGGATAAAGTTGCAGGACCACTTCTGCGCTCGGCCCTTCCGGCTGGCTGGTTTATTGCTGATAAATCTGGAGCCGGTGAGCGTGGGTCTCGCGGTATCATTGCAGCACTGGGGCCAGATGGTAAGCCCTCCCGTATCGTAGTTATCTACACGACGGGGAGTCAGGCAACTATGGATGAACGAAATAGACAGATCGCTGAGATAGGTGCCTCACTGATTAAGCATTGGTAACTGTCAGACCAAGTTTACTCATATATACTTTAGATTGATTTAAAACTTCATTTTTAATTTAAAAGGATCTAGGTGAAGATCCTTTTTGATAATCTCATGACCAAAATCCCTTAACGTGAGTTTTCGTTCCACTGAGCGTCAGACCCCGTAGAAAAGATCAAAGGATCTTCTTGAGATCCTTTTTTTCTGCGCGTAATCTGCTGCTTGCAAACAAAAAAACCACCGCTACCAGCGGTGGTTTGTTTGCCGGATCAAGAGCTACCAACTCTTTTTCCGAAGGTAACTGGCTTCAGCAGAGCGCAGATACCAAATACTGTCCTTCTAGTGTAGCCGTAGTTAGGCCACCACTTCAAGAACTCTGTAGCACCGCCTACATACCTCGCTCTGCTAATCCTGTTACCAGTGGCTGCTGCCAGTGGCGATAAGTCGTGTCTTACCGGGTTGGACTCAAGACGATAGTTACCGGATAAGGCGCAGCGGTCGGGCTGAACGGGGGGTTCGTGCACACAGCCCAGCTTGGAGCGAACGACCTACACCGAACTGAGATACCTACAGCGTGAGCTATGAGAAAGCGCCACGCTTCCCGAAGGGAGAAAGGCGGACAGGTATCCGGTAAGCGGCAGGGTCGGAACAGGAGAGCGCACGAGGGAGCTTCCAGGGGGAAACGCCTGGTATCTTTATAGTCCTGTCGGGTTTCGCCACCTCTGACTTGAGCGTCGATTTTTGTGATGCTCGTCAGGGGGGCGGAGCCTATGGAAAAACGCCAGCAACGCGGCCTTTTTACGGTTCCTGGCCTTTTGCTGGCCTTTTGCTCACATGTTCTTTCCTGCGTTATCCCCTGATTCTGTGGATAACCGTATTACCGCCTTTGAGTGAGCTGATACCGCTCGCCGCAGCCGAACGACCGAGCGCAGCGAGTCAGTGAGCGAGGAAGCAATTCgaatccgcgttttctccgtgaaagggaggtgtcctaggcctctagacgatgggggctttttgttatattttactaaatatatattataattaaaaaaaattgaattgtcaatttttaatgtacacttagttgaaagtgcccctgtccccttggccatatttaacagaagttatttataacgcagctgttttttggagtctataaatttataacatcagttactatggatttccctttagttttatggcctaggacgtccccttccccttcgatgctggaggcatccttttacgggacaataaataaatttgttgcctcgcctatcggctaacaagttccttcggagtatataaatataggatgttaatactgctataaactttagttgcccaatatttatattaggacgccagtggcagtggtaccgccactgcctgcttcgcagtatataaatataggcagttggcaggcaactgccactgacgtcctattttaatactcccaagtttacttgcctaggcagttggcaggcaacaaatttatttattgtccactaaaatttatttgcccgaaggggacgtccactaaaatttatttacccgaaggggacgtcctaatataaatatggggatgtcaatgctccgttaggaagtaactaacgtttttcaaataaattttatcccggagggaagtaggcagtagcccgccactgtcatcctttaagtggatctctcgtcaggcaatttgcttacacctttaaattaaaaattaaatttaaagaaaagtgagctattaACGCGTtaacccatgattaacaactatatcaataaaatcaatttgtagtgaaatactctga**ttgaca**ttaaaataataccatgataaaaat**tataat**aacaaattttacgtccttagttcagtcggtagaacgcaggttt**tca**aaacctgatgtcgtgggttcaattcctacagggcgtgtttttcctaatgtactttgttgtaaaagtggctggtttaacctttttaggtttcggattgaacaataatggcagttaagagtcactaaagctgctgtatagACGCGTaagctttcttaattcaacatttttaagtaaatactgtttaatgttatacttttacgaatacacatatggtaaaaaataaaacaatatctttaaaataagtaaaaataatttgtaaaccaataaaaaatatatttatggtataatataacatatgatgtaaaaaaaactatttgtctaatttaataaccatgcattttttatgaacacataataattaaaagcgttgctaatggtgtaaataatgtatttattaaattaaataattgttattataaggagaaatccATGggaagagctactccatggatcctctagagtcgacctgcaggcatgcaagcttgtactcaagctcgtaacgaaggtcgtgaccttgctcgtgaaggtggcgacgtaattcgttcagcttgtaaatggtctccagaacttgctgctgcatgtgaagtttggaaagaaattaaattcgaatttgatactattgacaaacttTAAtttttatttttcatgatgtttatgtgaatagcataaacatcgtttttatttttatggtgtttaggttaaatacctaaacatcattttacatttttaaaattaagttctaaagttatcttttgtttaaatttgcctgtctttataaattacgatgtgccagaaaaataaaatcttagctttttattatagaatttatctttatgtattatattttataagttataataaaagaaatagtaacatactaaagcggatgtaGCGCGTttatcttaacggaaggccagtggcagtggcggtgccactgccgaatataaatatggttgagttgcttagtttaccttagcgaaaagaagacttagcagctagccttaacaaacagttttatattttatgtttgtgttaaataaaaTTAAGAAACTTTAGCTAAAGTTTCCCAACTCATAGAAACGTCATCTAAAATTAAAGAACTGTTGTAAATTTCTAAAATGATTAATAAGAATGCTGCAAATAAAAGGATAAATACAGCCATTAAAACAGTTGTACCCCAGCCTGGTAATACTTTACCTGCTTCTGAGTTAAGTGGACGTAATAAAGTACCTAATGGTGTAACTAAACCAGGTTCTTGGAAGTCTGAATTTACTTTTGATGGTTTAGCTTTAGAAGTTCCTGTTGCCATaattgattaaatgaattaagcgttattagcgctattttatttactttctgtaaaaaataaggaaaatattcttcagtgcattccctctcaggattataaatactctgaggataacgttctctcgtcaaggggttgcttcttgtgagtatagaaacctactagcacaagaaataaattgcataaaaatgtatttacctaggaccgcagtaggcagtcccttttccccttcagaactgcctgctttaaaagaatgaaaaaactgccttgtctggtaagtaaaactctttaattactcactaaagacgatcttagaagttctttgttcattttttatttaatataatatttgttatataaaaattaaataatttttaattaatgtttaactttgtaaggacagtttcaaagtgacatgaatggctactgcaaaaacgaagtaagttattctttctcagggcaaaattttgagtagattaattttgtttaaaaatgtgggacacagtcgtcaagtcttttgaactatctaagagatatgttgaaaagagaataattttattattaaatgagctatggaaagtccagcttttttctttaccttttttttatggtttcttctgttaagtgtaactggctattcagtttatgttagttttggtccaccttcaaaaaaattacgtgatccttttgaagaacacgaagattaaacaagttaaaaagtactatttttacaagtgacttcggtgcctctgagaaccctagttatagtgatataaaataactagctaactactttatatttttatgaaagtcattttgtcgagcatataaacaaaaacaaaattgctatactaggcagtcacagtgcaactgtctccgtctccttaaccgagaaagggtaaacgtcttcggtaaagtaacaaactttagttatgttaactgcttgcgagttaaccattttttttcctccgaaggacaacagttggcagttgccaaactttagtggtctaatatttatattaggcagttggcaggcaactgcactgacgtcccgaaggggaaggggtttacttacctcctaacggagtatataaatagaataaaatttatttcctgcgctagcagatttacatactaggattttaatactccgaaggaggcagtggcggtaccactgccactggcgtcctccttccccttcgggcaaatgcattttagtgccacttaagtttacttgcctaggcagttggcaggacgtcagtggcagtggtaccgcgactgcctatatttatatactcctaagtttacttgcctaggcagttggcaggcaactgccactgacgtccttccccttccccttcgggacgtccccttacgggaatataaatattagtggatatttatatactgcgatgtttacatactccgaaggaggagagctagcagttgcctgccaactgcctaatataaatattgggcaagtaaacttagaatgtttacatactccgaaggaggacgtcccttacgggaatataaatattagtggcagtggtaccgccactgcctccttcggagtattaaaatcctagtatataatataccgtaagggacgtcctccgacggtggcagtggcggtaccactgccaccggcgtcctaatatacatattgaagtatttaaacctgttagcgcacgctctaacgagtcagtaaacttcccttttggggcttctaggcagcgcataaattttctaggacgaacgtccactggcgtctcgtaaggagcagtgacaggccactaatgtccccttaatgggtaaataaatggctatcgtctatccatgaagagaccatatattccagtagcaccgttatgatcctcaaagggtaacaccatttgtatagtattatggtgaaatgcatccctttcagggtagatttatatcttacaG
